# Light Intensity Modulates the Effect of Phosphate Limitation on Carbohydrates, Amino Acids, and Catechins in Tea Plants (*Camellia sinensis* L.)

**DOI:** 10.3389/fpls.2021.743781

**Published:** 2021-10-08

**Authors:** Santosh KC, Lizhi Long, Meiya Liu, Qunfeng Zhang, Jianyun Ruan

**Affiliations:** Key Laboratory of Tea Plant Biology and Resources Utilization (Ministry of Agriculture), Tea Research Institute, Chinese Academy of Agricultural Sciences, Hangzhou, China

**Keywords:** *Camellia sinensis* L, phosphorus, light intensity, metabolic pathway, gene expression, interaction

## Abstract

Metabolites are major contributors to the quality of tea that are regulated by various abiotic stresses. Light intensity and phosphorus (P) supply affect the metabolism of tea plants. However, how these two factors interact and mediate the metabolite levels in tea plants are not fully understood. The present study investigated the consequences of different light intensity and P regimes on the metabolism of carbohydrates, amino acids, and flavonoids in the Fengqing tea cultivar. The leaves and young shoots were subjected to untargeted metabolomics analysis by two-dimensional gas chromatography coupled to time-of-flight mass spectrometry (GC×GC–TOF/MS), ultra-performance liquid chromatography-quadrupole-TOF/MS (UPLC–Q–TOF/MS), and targeted analysis by high-performance liquid chromatography (HPLC) along with quantification of gene expression by quantitative real time-PCR (qRT–PCR). The results from young shoots showed that amino acids, pentose phosphate, and flavonol glycosides pathways were enhanced in response to decreasing light intensities and P deficiency. The expression of the genes *hexokinase 1, ribose 5-phosphate isomerase A (RPIA), glutamate synthetase 1* (*GS1), prolyl 4-hydroxylase (P4H)*, and *arginase* was induced by P limitation, thereafter affecting carbohydrates and amino acids metabolism, where shading modulated the responses of transcripts and corresponding metabolites caused by P deficiency. P deprivation repressed the expression of Pi transport, stress, sensing, and signaling *(SPX2)* and induced bidirectional sugar transporter *(SWEET3)* and amino acid permeases *(AAP)* which ultimately caused an increase in the amino acids: glutamate (Glu), proline (Pro), and arginine (Arg) under shading but decreased catechins [epicatechingallate (ECG) and Gallic acid, GA] content in young shoots.

## Brief Summary Statement

The interaction of light intensity and phosphorus availability at transcriptional and metabolic levels influenced amino acids, carbohydrates, and flavonoid homeostasis.

## Introduction

Tea metabolites are key components of taste, flavor, and health benefits. These metabolites are largely regulated by various environmental factors such as light intensity and phosphate (Pi) availability during plant growth and development. Light is essential to photosynthesis and other subsequent physiological processes in plants. Phosphorus (P) plays an enormous role in the regulation of whole plant growth and, more importantly, the quality of tea's young shoots. Despite abundant total P in soils, the availability of P in tea gardens is relatively low due to the fixation with iron and aluminum in the acidic soils (Shen et al., 2011). Therefore, Pi is one of the most important fertilizers for tea productivity and quality. In agricultural practice, shading is frequently applied in the management of tea gardens as it can improve the quality of green tea by decreasing the ratio of polyphenols to amino acids and reducing the levels of galloylated catechins in tea shoots (Zhang et al., 2021). Thus, the understanding of how P and light affects the metabolites of tea plants, and their potential interaction will contribute to balancing the yield and quality of tea in tea production.

There is profound evidence on the effects of P on plant growth and the contents of other nutrients in plant organs. P deficiency negatively impacts plant growth and productivity (Stewart et al., 2001). To adapt to low P, plants may respond by modifying the root architecture and morphology (Liao et al., 2004; Zhou et al., 2008; Tian et al., 2012). Specifically, by increasing the exudation of protons and organic acids (Fox and Comerford, 1990; Ström et al., 2005; Taghipour and Jalali, 2013), enhancing the secreted or root-associated acid phosphatase activities (del-Pozo et al., 1999; Bozzo et al., 2002; Ligaba et al., 2004; Wang et al., 2009, 2012; Liang et al., 2010; Robinson et al., 2012), reducing the synthesis of phosphorylated metabolites (Pant et al., 2015; Ding et al., 2017), or by decreasing the evapotranspiration from leaves (Motalebifard et al., 2013). Moreover, P limitation has been reported to decrease the water-extractable concentrations of total polyphenols and free amino acids in leaves (Lin et al., 2012). In particular, the contents of amino acids such as theanine (Thea), aspartic acid (Asp), and glutamic acid (Glu) were downregulated (Lin et al., 2012; Ding et al., 2017) upon P starvation. In contrast, P limitation increased the concentrations of water-soluble sugars, along with the amino acids valine (Val), proline (Pro), and cysteine (Cys), and the ratio of total polyphenols to total free amino acids (Lin et al., 2012).

The elevated light intensity has been reported to increase shoot photosynthetic carbon fixation and the supply of carbohydrates to root but to decrease P concentration in plants (Taylor et al., 2008; Cheng et al., 2014). The leaves of tea plants cultivated in the shade contain higher amino acid contents than those grown in full sunlight due to regulation by sugar supply and a variety of regulatory signals (Lam et al., 1996). On the one hand, light regulates photosynthetic carbon assimilation (Timm et al., 2013), and shading preserves amino acids from protein synthesis (Schlüter et al., 2013), thereby reserving energy for plants during periods of extended darkness (Gipson et al., 2017). On the other hand, light regulates the expression of genes which are also involved in low P responses *via* mediating the synthesis and transport of photosynthates (Hammond and White, 2008; Zhou et al., 2020).

Numerous genes involved in sugar biosynthesis have been reported to be regulated by light and mineral availability (Lejay et al., 2008; Granot et al., 2014). For instance, the crosstalk of sugars and Pi is mediated by *hexokinase (HXK)* which could be related to downstream steps of sugar metabolism *via* the HXK pathway involving glycolysis rather than sugar sensing (Granot et al., 2014). Pi starvation-induced genes, such as β*-AMY* and *CHS*, are also sugar-induced independently from the HXK pathway, suggesting an interaction between Pi starvation signaling and HXK-independent sugar sensing in Arabidopsis (Müller et al., 2005; Ceasar, 2020). In addition, genes related to carbohydrate anabolism and catabolism (Zhang et al., 2003; Quehenberger et al., 2019), run parallel to glycolysis (Kruger and von-Schaewen, 2003), regulate ammonium assimilation (Kusano et al., 2020) and polyamine levels (Majumdar et al., 2016), and inhibit flavonol biosynthesis through polymerization (Yin et al., 2012; Wang et al., 2018), have been reported. Amino acids are responsible for tea quality. Low-quality grades of black tea were found to contain lower free amino acid contents (Alasalvar et al., 2012). In tea, the most abundant amino acids are Thea, Gln, Glu, and Arg (Ruan et al., 2012). The coefficient values in one study revealed that Gln, Arg, and Thea were the major contributors to high-ranking tea, whereas Asp and Ala were the main components in low-ranking tea (Miyauchi et al., 2014).

The biosynthesis of metabolites in the organs of tea plants responds differentially to variable light intensities and P supply, leading to specific changes in metabolite levels. On the one hand, the expression of genes for enzymes and transcription factors drives the synthesis of metabolites. On the other hand, the metabolites could regulate the expression and functionality of the genes by feedback loops. Thus, understanding these molecular networks on carbohydrates, amino acids, and flavonoids is a prerequisite to optimizing the effects of the related abiotic factors. Untargeted metabolomics profiling based on two dimensional gas chromatography coupled to time-of-flight mass spectrometry (GC×GC–TOF/MS) and ultra-performance liquid chromatography-quadrupole-TOF/MS (UPLC–Q–TOF/MS), integrated with targeted amino acid and catechins by high-performance liquid chromatography (HPLC) were used to investigate the effects on metabolites in mature leaves, roots, and young shoots of tea plants under different light and P levels. To gain a deep understanding of the molecular mechanisms under specific pathways, the expression of transcription factors and genes for metabolic biosynthesis was investigated in parallel with metabolite analysis. The objective of this study was to gain a systematic understanding of the responsive pathways, their specific metabolites, and expression of genes to light intensity, P, and their interactional effects on tea quality components.

## Materials and Methods

### Plant Materials

The tea cultivar Fengqing was planted in pots at the Tea Research Institute, Hangzhou. The soil growth medium was prepared from thoroughly mixed pertile, vermiculite, and turf with volume in the ratio of 1:2:3 (1:2:3, v/v). Coarse sand was mixed thoroughly with the growth medium (1:1, w/w) to make it stable. The plants were exposed to full natural light (100%; FL), medium light (50% of full light; ML), and low light (20% of full light; LL) by shading with black perforated nylon agronet and measuring the light intensity in the plant vicinity using a LUX meter. The light intensity was measured weekly. The control (+P) treatment was applied by adding 0.1 mM of P as KH_2_PO_4_, while no P was added in the low (–P) treatment. Each treatment included four pots and each pot contained four plants. Plants were irrigated with an automatic micro-sprinkler system every day to ensure an adequate supply of water.

Plants with uniform height and biomass were used for the experiment and sampling started after 1 year from stably growing plants. Young shoots (a bud with the first leaf) and leaves (fifth leaf) were harvested six times (*n* = 6; three times for each in two independent experiments) during 14 weeks period. The samples were collected on the same day at the same time (9:30 am) within a range of (10 min) to avoid differences caused by temperature, relative humidity, and light. Materials collected for analysis were divided into two parts: one part was collected and frozen immediately in liquid nitrogen, and stored at −80°C, whereas the other part was dried to be used for elemental analysis. After collecting shoot samples, the remaining part of each plant was pulled out from the soil, washed carefully with de-ionized water, and the fresh fibrous root was separated, frozen in liquid nitrogen, and stored at −80°C for further analysis.

### Processing of the Samples and Instrument Conditions for Untargeted Metabolomics

The frozen young shoots, leaves, and roots were ground into a fine powder using liquid nitrogen. About 100 g of each sample was ground and exactly weighed in a 2 ml tube under chilled conditions using ice cubicles and liquid nitrogen. For metabolomic analysis based on UPLC-Q-TOF/MS, the plant samples were extracted with 2 ml of a solvent mixture of 75% methanol and 1% formic acid for 10 min in an ultrasonic bath and then centrifuged at 12,000 rpm for 15 min at −4°C. Two milliliters of extracted samples were filtered through a 0.22 mm PTFE filter and injected into a 1 dram glass vessel. GC × GC–TOF/MS samples were extracted using 1,000 ml of methanol-chloroform (3:1, v/v) solvent (Sigma-Aldrich Co., St. Louis, MO, USA) from the plant sample. Ten milliliters of L-2-chlorophenyl alanine (0.2 mg/ml in water) were mixed by vortexing (30 s) and then centrifuged at 12,000 rpm for 10 min at −4°C. The supernatant (400 ml) was dried with vacuum centrifugation without heating. The samples were frozen in liquid nitrogen and 80 ml of methoxyamine was added (8 mg/ml in pyridine) to each dried sample followed by vortexing for 1 min. Methoxymation and trimethylsilylation were performed as described previously (KC et al., 2018) and the glass vials were sealed for sample analysis.

The machine consisted of an Agilent GC 6890N gas chromatograph, the high-speed TOF mass spectrometer detector (Pegasus HT, Leco Co., CA, USA). It was equipped with DB-5 MS column (30 m × 250 μm i.d., 0.25 μm film thickness) as the first dimension column and a DB-17H (2.5 m × 0.1 mm I.D., 0.1 μm film thickness) as the second-dimension column. Helium was used as carrier gas at a constant flow rate of 1 m min^−1^. The GC oven temperature was initially set at 80°C for 2 min, then raised to 180°C at a rate of 10–240°C, at a rate of 5–290°C, and finally held at 290°C for 9 min. The secondary oven temperature was kept at 5°C offset above the primary oven. Each 1 μl aliquot of the derivatized sample was injected in splitless mode, and the injector temperature was set at 270°C. The temperatures of the transfer line and ion source were set at 260 and 200°C, respectively. The mass spectrometry data were acquired with electron impact ionization (70 eV) at full scan mode (m/z 30–600). The dwelling time for each scan was set at a rate of 20 spectra per second and the solvent delay at 3 min.

The metabolites were achieved in the ultra-performance liquid chromatography (ACQUITY UPLC, Waters Corp., Milford, MA), which was equipped with an Acquity HSS T 3 column (1.8 mm, 100 mm, 62.1 mm; Milford, MA, USA) kept at 40°C, and connected to a quadrupole-time of flight mass spectrometer (Xevo G2-XS QTOF, Waters Corp.). Mobile phase A was 0.1% aqueous formic acid, and mobile phase B was acetonitrile containing 0.1% formic acid. Gradient elution with a flow rate of 0.4 ml/min was performed as follows: the gradient profile was 4% B from 0 to 1 min, 4–7% B at 1–3 min, 7–25% B at 3–5 min, 25–45% B at 5–8 min, 45–90% B at 8–10 min, 90–100% B at 10–12 min, and 100% B at 12–15 min. The injection volume of the samples was 2 μl, and the samples were kept at 6°C during analysis. After each injection, the needle was rinsed with 600 μl of weak wash solution (water/methanol, 90:10) and 200 μl of strong wash solution (methanol/water, 90:10). Mass spectra were acquired using electrospray ionization at positive and negative modes over the range of m/z 100–170. The stability of the method was tested by performing 10 repeated injections of the mixed samples every 2 h. The maximal tolerated m/z deviation, minimum or maximum chromatographic peak width in consecutive scans, and allowable retention time deviations were set as 15 ppm, 5/20 s, and 2 s, respectively.

### Quantifying Targeted Catechin and Amino Acids by HPLC

Samples for catechin determination were treated as described for UPLC–Q–TOF/MS (KC et al., 2018). For free amino acid analysis, samples were derivatized as recommended by the waters AccQ Tag chemistry package. HPLC analysis was carried out using an e2695 connecting 2,998 photodiode array detector system (Waters Corp., Milford, MA, USA) injected with 10 or 25 μl of sample solutions for catechin or free amino acid, respectively. For catechins, distilled water with 2% formic acid was used as mobile phase A. Mobile phase B consisted of HPLC solvent Acetonitrile (ACN). The samples were eluted at column temperature 40 ± 1°C at a flow rate of 1 ml/min and monitored at 278 nm. Similarly, for amino acids, the AccQTag eluent from waters was used as mobile phase A. Mobile phase B was ACN, and the column temperature was set at 37 ± 2°C. The procedure was completed as prescribed in the AccQ Tag chemistry package instruction manual. Peaks of catechins were identified by comparing the retention times of the samples to those of authentic standards and amino acids were identified as prescribed in the manual.

### qRT–PCR and ICP AES Analysis

The extracted RNA was used to synthesize cDNA using PrimeScript Reagent Kit. Transcripts of each gene were quantified as cp value using Light Cycler 480 II real-time machine with Light Cycler 480 Software version 4.0 (Roche, Mannheim, Germany; Singh et al., 2015). To integrate the corresponding changes, four different P responsive genes and 19 metabolic genes from different pathways were selected and used to manifest the response to P treatments under different light intensities. The primer sequences of the genes used for qRT-PCR are shown in Supplementary Tables 1, 2 and the reference gene *GAPDH* with sample (FL+P) was used for normalization (Xiong et al., 2013). The P concentrations in freeze-dried plant samples were measured by ICP–AES following digestion with concentrated HNO_3_ and HClO_4_ (Salahinejad and Aflaki, 2010).

### Data Processing, Analysis, and Visualization

The data were collected from two independent experiments with three biological replicates each. The whole metabolomics data were screened and identification was performed prior to normalizing the data. Data files from GC × GC-TOF/MS were processed in Leco software (Leco Co., St. Joseph, MI, USA) and were deconvoluted using Automatic Mass spectral Deconvolution and Identification Software (AMDIS). The corresponding peaks of each chromatogram were compared to the National Institute of Standards and Technology (NIST, FairCom Co., Gaithersburg, MD, USA) mass spectral database to identify metabolites. Raw chromatographic data acquired from the UPLC-Q-TOF/MS analysis were processed by TransOmics (Waters Corp., Milford, MA, USA), and peaks were annotated from the accurate mass measurements using online metabolite databases. The metabolites were identified based on actual mass, retention time, and isotopic distribution, and accurate mass measurements were confirmed from Metlin online web-based database and our published literature (KC et al., 2018).

After normalization (a relative expression to FL+P), PCA analysis was performed using the R package “MetaboAnalystR” on samples from a different light and P treatments to observe for PLS–DA test for separation of the group (Supplementary Figure 1) and data analysis for the enriched pathways and pathway impact. Similarly, the data from HPLC and ICP–AES were used to measure the contents of targeted metabolites and nutrition content in young shoots and leaves under different light and P treatments. Furthermore, all the spectrometric measurements were analyzed with two–way ANOVA to compare means of single light effect, P effect and their interaction, and one-way ANOVA to compare means of full light (FL), medium light (ML), low light (LL), and shading effect (FL × ML and LL) using Tukey's HSD with R package “agricolae.” The enriched pathways, pathway impact, pathway map, bar plot, box plot, and circular plot were drawn using the latex “tkiz” package. Pearson's correlations were performed using the R statistical package “pcorr.” The linear regression graph and bi-plot PCA figure and heatmap figure were drawn collectively using the R and Latex program.

## Results

### Plant Biomass and Element Concentrations

The dry weights of all tissues were significantly decreased under P deficiency in ML (50% of full light) conditions (Table 1; Supplementary Figure 2). The shading treatment increased the total biomass of young shoots, leaves, and roots in ML compared to FL and LL with P sufficient conditions whereas the total biomass in LL was decreased compared to FL and ML under P-deficient conditions. In contrast, both 50% (ML) and 80% (LL) shading treatments did not significantly affect the total biomass of young shoots, leaves, and roots under P deficiency. Thus, the interaction between light regimes and P levels was observed in terms of the biomass of young shoots and leaves. The P concentrations in young shoots and leaves decreased under P deficiency regardless of the light intensity. Upon decreasing the light intensities from FL to LL, P concentration in young shoots was reduced under both P-sufficient and P-deficient conditions (Table 1; Supplementary Figure 3).

**Table 1:** Biomass and concentrations of phosphorus (P) in the organs of Fengqing cultivar under different light intensities and phosphorous (P) levels.

**Parameter**	**Organ**	**P level**	**Light intensity**			**Significance**		
			**FL**	**ML**	**LL**	**Light**	***P***	**L × P**
Biomass	Young Shoots	+P	6.23 ± 0.82*b*	8.11 ± 1.73*a*	6.15 ± 0.77*b*	*p* < 0.01	*p* < 0.001	*p* < 0.01
(g plant^−1^)		−P	3.48 ± 0.48*c*	4.1 ± 0..31*c*	5 ± 0.78*bc*			
	Leaves	+P	4.72 ± 0.89*ab*	6.06 ± 0.61*a*	3.86 ± 0.93*b*	*p* < 0.01	*p* < 0.001	*p* < 0.01
		−P	3.71 ± 0.48*b*	3.88 ± 0.98*b*	3.97 ± 0.85*b*			
	Root	+P	1.73 ± 1.81*ab*	12.48 ± 2.85*a*	7.67 ± 1.13*bc*	*p* < 0.001	*p* < 0.01	ns
		−P	9.97 ± 1.4*ab*	9.07 ± 2.42*b*	5.43 ± 0.55*c*			
	Total	+P	21.68 ± 1.97*b*	26.66 ± 3.75*a*	17.68 ± 1.26*bc*	*p* < 0.001	*p* < 0.001	*p* < 0.01
		−P	17.16 ± 1.42*c*	17.04 ± 3.08*c*	14.41 ± 1.34*c*			
P (mg g^−1^)	Young Shoots	+P	9.57 ± 0.09*a*	6.52 ± 0.15*c*	4.76 ± 0.06*d*	*p* < 0.001	*p* < 0.001	*p* < 0.001
		−P	7.62 ± 0.04*b*	4.24 ± 0.08*e*	2.49 ± 0.02*f*			
	Leaves	+P	9.08 ± 0.34*a*	4.2 ± 0.22*d*	5.1 ± 0.2*c*	*p* < 0.001	*p* < 0.001	*p* < 0.001
		−P	6.87 ± 0.21*b*	3.29 ± 0.12*e*	3.44 ± 0.3*e*			

*Means with different letters in the same row of the same parameter and organ are significantly different. ns, non-significant differences, between light and P interaction*.

The shading treatments also affected the concentrations of other elements in P-sufficient and P-deficient plants (Table 2; Supplementary Figure 3). In young shoots, the concentrations of K, Mg, S, Ca, Cu, Fe, B, and Al decreased but those of Mn and Zn increased under shading. On the other hand, P limitation decreased the concentrations of Mg, Ca, Cu, Zn, Mn, and Al in young shoots under FL but increased S concentration regardless of light intensity. Similarly, S concentration decreased in young shoots under shading but increased in leaves under P limitation regardless of the light intensity. Taken together, the concentrations of elements, except Mn and Zn, decreased under shading and further decreased due to P deficiency in young shoots and leaves (Table 2; Supplementary Figure 3).

**Table 2:** Concentrations of macronutrients (K, Mg, Ca, S, and Al, mg g^−1^) and micronutrients (Zn, Mn, Cu, Fe, B, mg kg^−1^) in young shoots under different light intensities and P levels.

**Nutrient**	**P level**	**Light intensity**			**Significance**		
		**FL**	**ML**	**LL**	**Light**	***P***	**L × P**
K	+P	23.09 ± 1.31*a*	16.95 ± 1.48*b*	15.59 ± 0.53*b*	*p* < 0.001	*p* < 0.001	*p* < 0.001
	−P	22.74 ± 0.88*a*	16.3 ± 1.28*b*	11.19 ± 0.62*c*			
Mg	+P	2.49 ± 0.05*a*	1.4 ± 0.02*c*	1.07 ± 0.01*d*	*p* < 0.001	*p* < 0.001	*p* < 0.001
	−P	1.74 ± 0.04*b*	1.39 ± 0.02*c*	0.92 ± 0.03*e*			
Ca	+P	1.43 ± 0.03*a*	1.37 ± 0.02*b*	0.92 ± 0.02*d*	*p* < 0.001	*p* < 0.001	*p* < 0.001
	−P	1.33 ± 0.02*b*	1.04 ± 0.03*c*	0.9 ± 0.01*d*			
S	+P	1.9 ± 0.03*b*	1.44 ± 0*d*	0.88 ± 0.01*f*	*p* < 0.001	*p* < 0.001	*p* < 0.001
	−P	2.09 ± 0.01*a*	1.51 ± 0.03*c*	1.36 ± 0*e*			
Al	+P	0.35 ± 0.01*a*	0.25 ± 0.01*b*	0.23 ± 0*c*	*p* < 0.001	*p* < 0.0001	*p* < 0.001
	−P	0.26 ± 0.02*b*	0.25 ± 0*bc*	0.2 ± 0*d*			
Zn	+P	31.17 ± 3.07*c*	48.84 ± 0.18*b*	51.62 ± 1.34*a*	*p* < 0.001	*p* < 0.001	*p* < 0.01
	−P	26.36 ± 0.29*d*	48.19 ± 0.48*b*	49.27 ± 0.11*ab*			
Mn	+P	671.82 ± 1.37*d*	775.74 ± 7.81*c*	915.61 ± 49.1*a*	*p* < 0.001	*p* < 0.001	*p* < 0.001
	−P	457.8 ± 7.81*e*	765.07 ± 14.92*c*	859.43 ± 23.93*b*			
Cu	+P	23.48 ± 0.18*a*	19.51 ± 0.36*c*	18.9 ± 0.21*c*	*p* < 0.001	*p* < 0.01	*p* < 0.001
	−P	22.16 ± 0.72*b*	19.09 ± 0.81*c*	14.91 ± 0.53*d*			
Fe	+P	144.15 ± 3.38*a*	138.71 ± 4.14*a*	111.89 ± 4.26*c*	*p* < 0.001	*p* < 0.001	*p* < 0.001
	−P	14.52 ± 0.96*a*	126 ± 2.01*b*	88.74 ± 12*d*			
B	+P	42.79 ± 1.13*a*	35.51 ± 2.01*b*	32.48 ± 0.99*c*	*p* < 0.001	*p* < 0.01	*p* < 0.001
	−P	42.79 ± 0.44*a*	35.62 ± 1.74*b*	28.62 ± 0.28*d*			

*Means with different letters in the same row of the same parameter and organ are significantly different. ns, non-significant differences, between light and P interaction*.

### Comparative Effects of Different Light and P Levels on Untargeted Metabolites

#### Young Shoots

**Figure 1:**
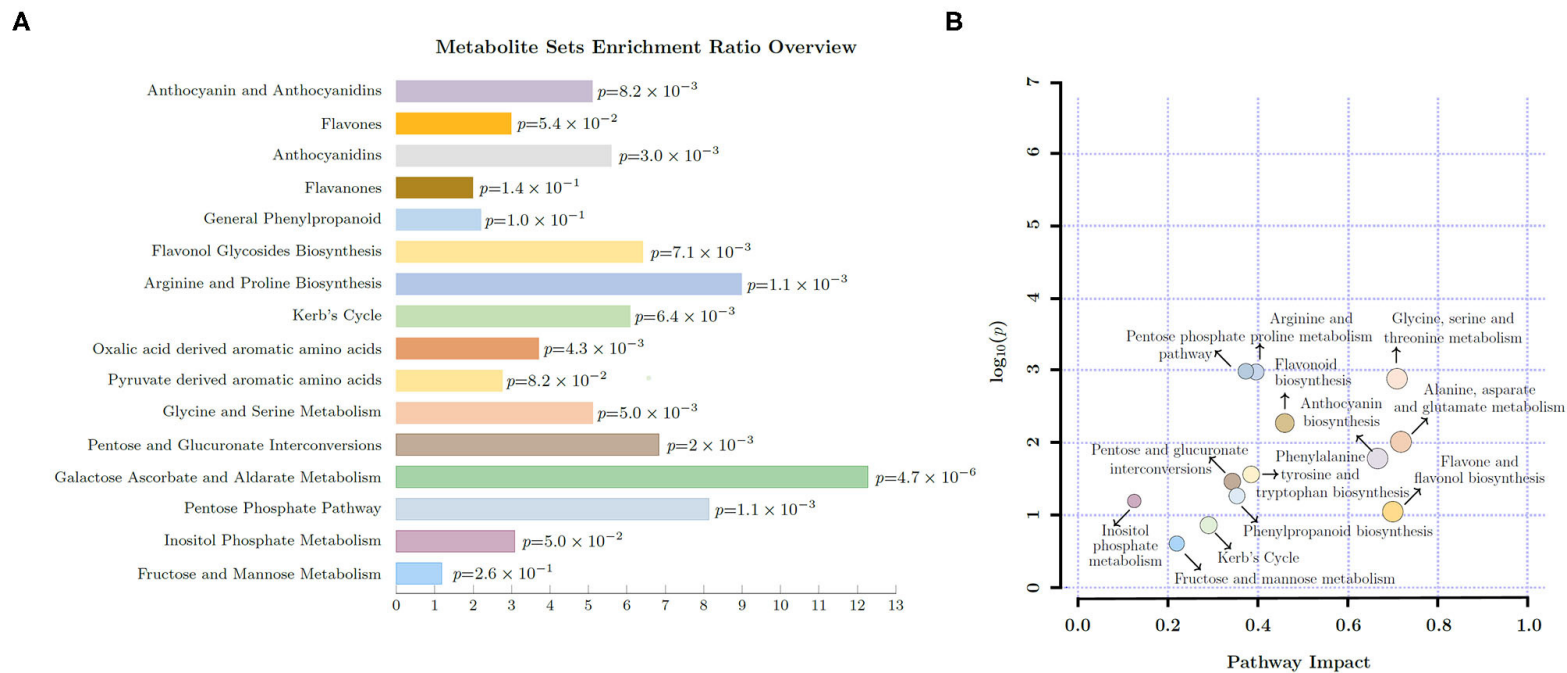
Overview of enriched pathways **(A)** and pathway impact **(B)** in young shoots of Fengqing cultivar exposed to different light intensities and Phosphorous (P) levels.

The metabolite sets enrichment ratio (MSEA) and pathway impact (PI) in young shoots were influenced by light intensities and P supply (Figure 1). The carbohydrate related pathways including pentose phosphate pathway (MSEA = 8.14; PI = 0.37), inositol phosphate metabolism (MSEA = 3.08; PI = 0.13), and fructose and mannose metabolism (MSEA = 1.18; PI = 0.22) were affected. A similar impact was noticed on amino acid pathways, viz., phenylalanine metabolism (MSEA = 2.21; PI = 0.47), TCA cycle (MSEA = 6.09; PI = 0.29), alanine, aspartate, and glutamate metabolism (MSEA = 3.7; PI = 0.72), and arginine and proline metabolism (MSEA = 8.98; PI = 0.40). Similarly, pathways related to secondary metabolites including flavone and flavonol biosynthesis (PI = 0.7) and flavonoid biosynthesis (PI = 0.46) were also greatly influenced by light intensity and P levels.

**Figure 2:**
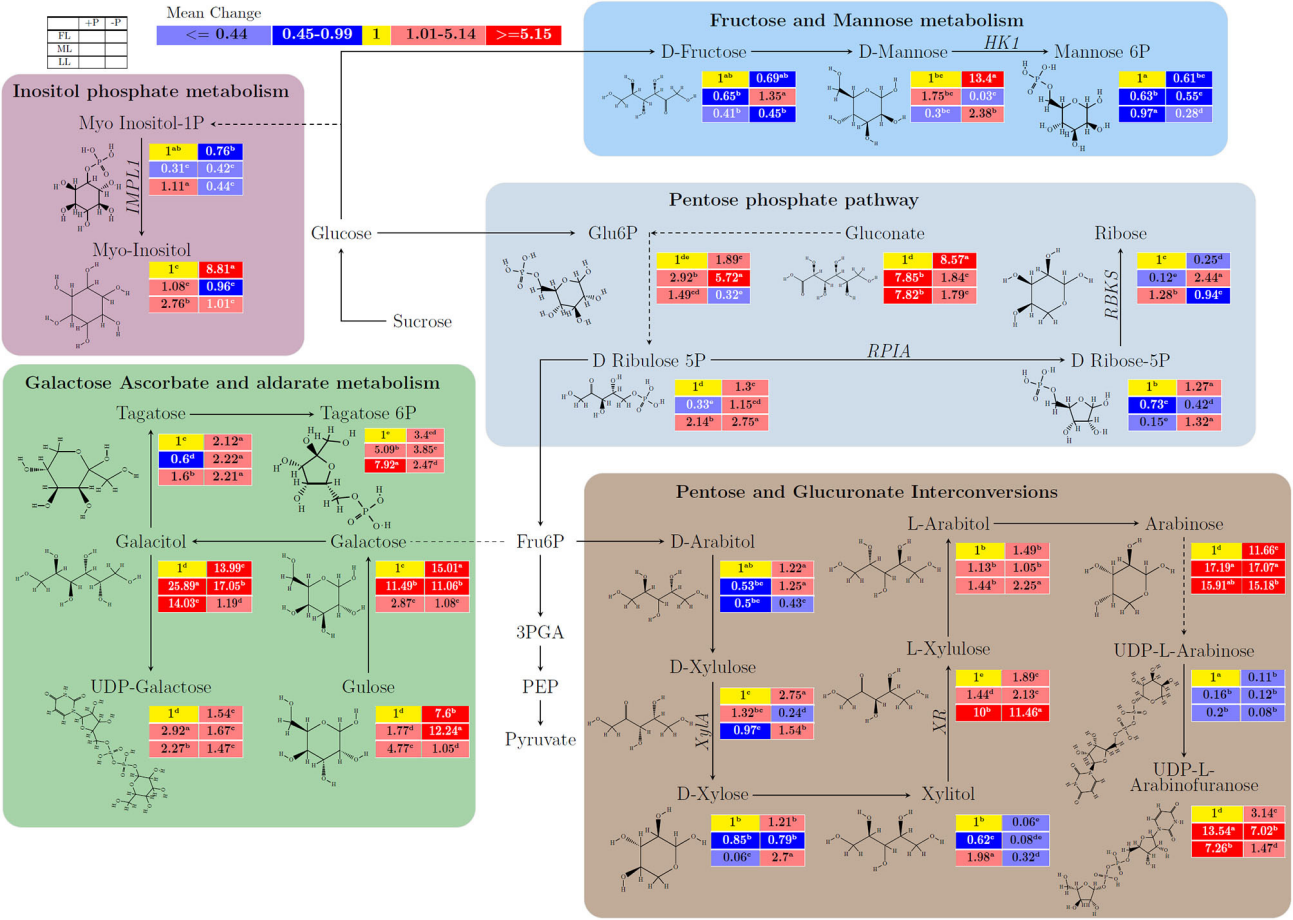
The light and P interactive effect on the biosynthesis of carbohydrates and/or related anaplerotic pathway metabolites to carbohydrates in young shoots of Fengqing cultivar measured by two-dimensional gas chromatography coupled to time-of-flight mass spectrometry (GC×GC–TOF/MS). The mean of each metabolite followed by a different letter in the heatmap table indicates a significant difference. In the heatmap, red boxes represent the value >1 and are separated into dark red and mild red by the mean value of the figured pathway data (>1) and subsequently <1 with blue color and mean value separated into mild blue and dark blue from figured pathway data (<1). The yellow box represents the normalization value. The blank table in the legend shows the position of the mean P and light treatment value of the metabolites.

In Figure 2 and Supplementary Table 3, the untargeted metabolites with respect to carbohydrates or anaplerotic pathways were detected in young shoots in response to different light intensities and P supply. In young shoots, within the fructose and mannose metabolism, D-mannose increased remarkably by P limitation from 1 to 13.4 (*p* < 0.001) in FL, and from 0.3 to 2.38 (*p* < 0.001) in LL, whereas mannose-6P decreased by –P treatment from 1 to 0.61 (*p* < 0.001) and from 0.97 to 0.28 (*p* < 0.001) under FL and LL conditions, respectively. Fructose and mannose metabolism related metabolites were responsive to sole light, P, and their interaction effect. In inositol phosphate metabolism pathway, myoinositol−1P was reduced by P limitation from 1 to 0.76 (*p* < 0.01) in FL and 1.11 to 0.44 (*p* < 0.001) in LL. By contrast, Myo–Inositol was increased from 1 to 2.76 (*p* < 0.001) by P supply under LL and raised from 1 to 8.81 (*p* < 0.001) by P deficiency under FL condition. In the pentose phosphate pathway, there was an opposite effect of shading on gluconate under P sufficiency and deficiency. On the other hand, P limitation increased gluconate from 1 to 8.57 (*p* < 0.001) in FL, whereas it decreased gluconate from 7.85 to 1.84 (*p* < 0.001) in ML and from 7.82 to 1.79 (*p* < 0.001) in LL. D–ribulose−5P increased significantly by P limitation regardless of light intensities. P deficiency increased D–ribose−5P but decreased ribose under FL and LL conditions. In addition, the light intensities affected the metabolic changes induced by P limitation in galactose ascorbate and aldarate metabolism (Figure 2). The interaction between light intensity and P supply was also observed in pentose and glucuronate interconversions (Supplementary Table 3).

**Figure 3:**
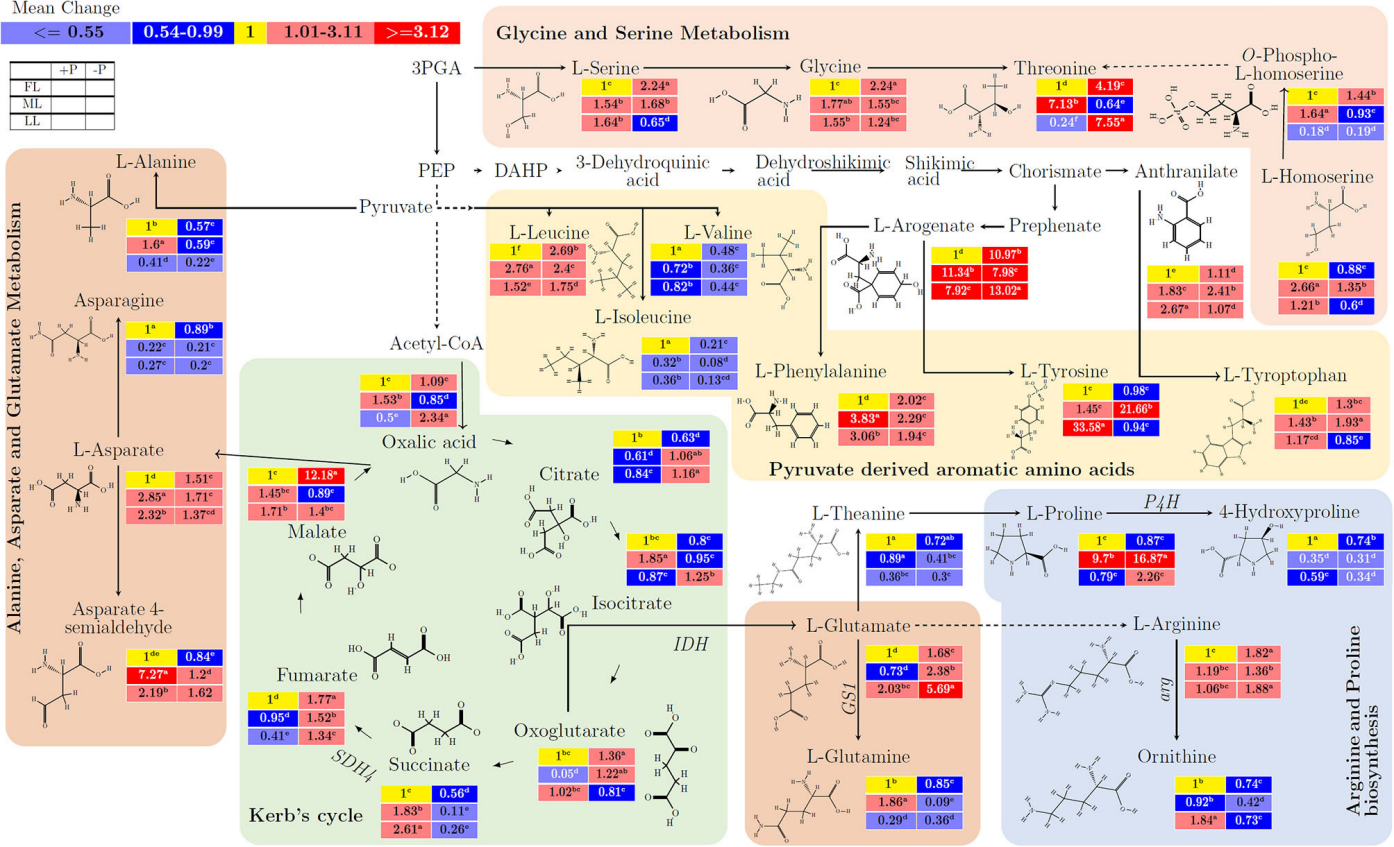
The interactive effect of light and P on the biosynthesis of amino acids and/or related anaplerotic pathway metabolites in young shoots of Fengqing cultivar measured by GC×GC–TOF/MS. The mean of each metabolite followed by a different letter in the heatmap table indicates a significant change in light and P treatments. In the heatmap, red boxes represent the value >1 and are separated into dark red and mild red by the mean value of the figured pathway data (>1) and subsequently <1 with blue and mean value separated into mild blue and dark blue from figured pathway data (<1). The yellow box represents the normalization value. The blank table in the legend shows the position of the mean P and light treatment value of the metabolites.

In glycine and threonine metabolism, there was a mean increase in L-serine (from 1 to 2.24; *p* < 0.001), glycine (from 1 to 2.24; *p* < 0.001), threonine (from 1 to 4.19; *p* < 0.001), and *O*–phospho–L–homoserine (from 1 to 1.44; *p* < 0.001) by P deficiency in FL (Figure 3; Supplementary Table 3). In the same pathway, there was no decrease in *O*–phospho–L–homoserine (from 0.19 to 0.18; *p* > 0.005) under LL condition. The pyruvate-derived amino acids, L–isoleucine (from 0.21 to 0.08; *p* < 0.001) and L–valine (from 0.48 to 0.36; *p* < 0.001) decreased due to P limitation. L–tyrsone significantly increased (from 1 to 33.58) by LL in P-sufficient condition but decreased (from 33.58 to 0.94) by P deficiency under LL (*p* < 0.001). In alanine, asparate, and glutamate metabolism, L–alanine (from 0.59 to 0.22; *p* < 0.001), L–asparagine (from 0.89 to 0.2; *p* < 0.001), and asparate 4–semialdehye (from 1.62 to 0.84; *p* < 0.001) decreased due to P limitation. There was a decrease in L–asparagine (from 0.27 to 0.2; *p* < 0.001) and increase in asparate 4–semialdehyde (from 1.62 to 7.27; *p* < 0.01) under shading condition. L–glutamate increased (from 1.68 to 5.69; *p* < 0.001) due to P limitation under all light conditions, but L–glutamate (from 0.29 to 0.36; *p* < 0.001) showed only mild increase under LL at –P condition. Oxalic acid (from 0.5 to 2.34; *p* < 0.001), citrate (from 0.84 to 1.16; *p* < 0.001), and isocitrate (from 0.87 to 1.25; *p* < 0.001) increased under LL at –P condition in Kerb's cycle. Similarly, succinate (from 0.56 to 0.11; *p* < 0.001) decreased while fumarate (from 1.34 to 1.77; *p* < 0.001) increased in comparison between P deficiency and P supply under all light treatments. In arginine and proline biosynthesis pathways, under shading condition, L–proline (from 0.87 to 16.87; *p* > 0.05) insignificantly increased whereas 4–hydroxyproline (from 0.74 to 0.31; *p* < 0.001) significantly decreased in comparison between P deficiency and P supply. In arginine and proline biosynthesis pathways with all light treatments, L–arginine (from 1.36 to 1.88; *p* < 0.05) increased but ornithine (from 0.74 to 0.42; *p* < 0.001) decreased in comparison between P deficiency and P supply.

**Figure 4:**
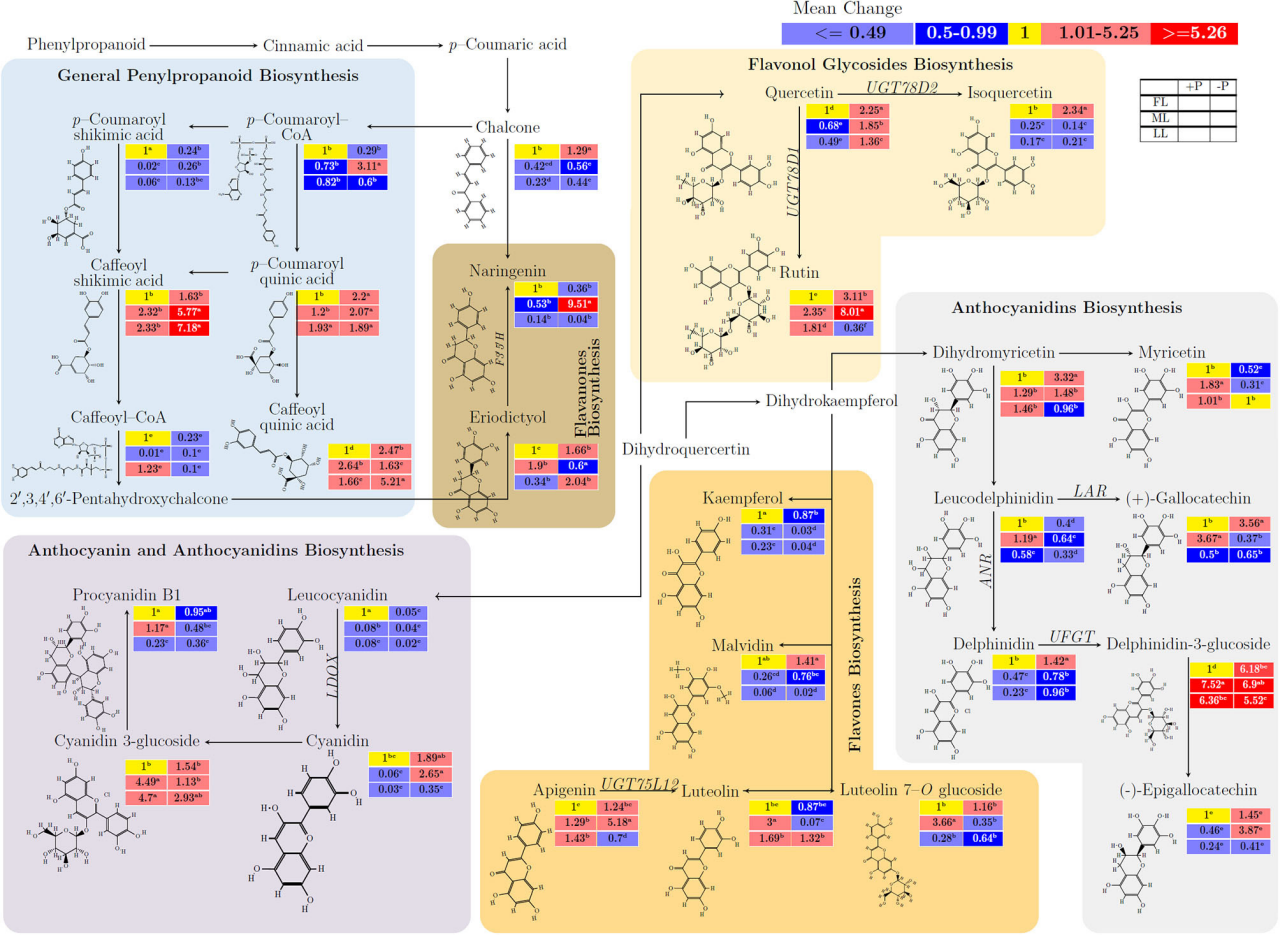
The interactive effects of light and P on the biosynthesis of flavonoids and/or related anaplerotic pathway metabolites of flavonoids in young shoots of Fengqing cultivar measured by ultra-performance liquid chromatography-quadrupole-time of flight mass spectrometry (UPLC–Q–TOF/MS). The mean of each metabolite followed by a different letter in the heatmap table indicates significant change due to light and P treatments. In the heatmap, red boxes represent the value >1 and are separated into dark red and mild red by the mean value of the figured pathway data (>1) and subsequently <1 with blue color and mean value separated into mild blue and dark blue from figured pathway data (<1). The yellow box represents the normalization value. The blank table in the legend shows the position of the mean P and light treatment value of the metabolites.

Figure 4 and Supplementary Table 4 show the influence of untargeted flavonoids with respect to light intensity and P supply in young shoots. There was a mean increase in quercertin (from 1 to 2.25; *p* < 0.001), isoquercertin (from 1 to 2.34; *p* < 0.001), and rutin (from 1 to 3.11; *p* < 0.001) in FL at –P but isoquerertin decreased (from 0.25 to 0.14; *p* < 0.001) due to shading conditions. Caffeoylshikimic acid (from 1.63 to 7.18) increased due to P deficiency (*p* < 0.001) and shading (*p* < 0.001). In flavanones biosynthesis pathways, there was an opposite response of narigenin and eriodictyol to P effect, where narigenin decreased while eriodictyol increased or vice versa. The anthocyanidin biosynthesis metabolite, leucodelphinidin (from 0.64 to 0.33; *p* < 0.001) decreased due to P limitation. Similarly, delphinidin (from 0.96 to 0.23; *p* < 0.001) decreased due to shading. There was a consistent decrease in the flavones biosynthesis-related metabolites kaempferol (from 0.31 to 0.04; *p* < 0.001) and malvidin (from 0.76 to 0.02; *p* < 0.001) due to shading. The P limitation decreased kaempferol (from 0.87 to 0.03; *p* < 0.001) and luteolin (from 1.32 to 0.07; *p* < 0.001) regardless of light intensity. In anthocyanidin and anthocyanidins biosynthesis pathways, leucocyanidin decreased (from 0.05 to 0.02; *p* < 0.001), and cyanidin increased (from 0.35 to 2.65; *p* < 0.001) due to P-deficient conditions in comparison between P deficiency and P supply under all light treatments. There was a decrease in leucocyanidin (from 0.08 to 0.02; *p* < 0.001) and cyanidin 3–glucoside (4.7 to 2.93; *p* > 0.05) whereas cyanidin (from 0.03 to 0.35; *p* < 0.05) and procyanidin B1 (from 0.23 to 0.36; *p* < 0.05) increased under LL at P-deficient conditions.

#### Leaves

In leaves, Myo inositol−1P increased (from 1.14 to 1.54; *p* < 0.01) and Myo–inositol decreased insignificantly (from 0.97 to 0.66; *p* > 0.05) under ML due to P limitation. D-ribulose (from 1 to 0.51; *p* < 0.001), D-ribose-5P (from 1 to 0.96; *p* < 0.001), and ribose (from 1 to 0.58; *p* < 0.001) decreased under FL at –P condition (Supplementary Table 3). Low light increased D–ribulose (from 0.55 to 0.62; *p* < 0.001), D–ribose-5P (from 0.18 to 1.31; *p* < 0.001) but decreased ribose (from 2.46 to 1.27; *p* < 0.001) in comparison between P deficiency and P supply. Limitation of P increased D–xylulose (from 2.23 to 7.14; *p* < 0.001) and D–xylose (from 1.58 to 4.04; *p* < 0.001) but decreased xylitol (from 2.23 to 0.11; *p* < 0.001) and L-xylulose (from 3.16 to 0.88; *p* < 0.05) under FL condition. ML decreased D–xylulose (from 7.14 to 2.23; *p* < 0.001), D–xylose (from 4.04 to 1.05; *p* < 0.01), and xylitol (2.23 to 1.67; *p* < 0.001) but increased L–xylulose (1.61 to 3.16; *p* < 0.001) compared with other light treatments due to P limitation. L–glutamate (from 0.7 to 0.35) and L–glutamine (from 0.76 to 0.2) decreased significantly in FL and ML. However, they increased under LL due to P-deficient conditions. Under shading, succinate (from 0.43 to 1.47; *p* < 0.001) and fumaric acid (from 1.21 to 1.49; *p* < 0.001) increased in ML but decreased significantly in LL due to P limitation. L–proline (from 1.51 to 0.68; *p* < 0.001) and hydroxyproline (from 4.44 to 1.57; *p* < 0.001) decreased in LL due to P limitation. Shading increased quercetin (from 0.52 to 0.62; *p* < 0.001) but decreased isoquercitrin (from 1.48 to 1.42; *p* > 0.05) and rutin (from 1.52 to 1.19; *p* < 0.01) due to P limitation (Supplementary Table 4). Leucodelphinidin (from 1 to 1.18; *p* < 0.001) and delphinidin (from 1 to 5.66; *p* < 0.001) increased but myricetin (from 1 to 0.45; *p* < 0.001), (+)–gallocatechin (from 1 to 0.21; *p* < 0.001), and delphinidin 3–glucoside (from 1 to 0.68; *p* < 0.001) decreased under FL at –P condition. In ML, (+)–gallocatechin (from 0.4 to 0.54; *p* < 0.001) and delphinidin (from 3.17 to 4.6; *p* < 0.001) increased, but myricetin (from 0.14 to 0.82; *p* < 0.001), leucodelphinidin (from 0.93 to 0.84; *p* < 0.01), and delphinidin 3–glucoside (from 0.99 to 0.76; *p* < 0.001) decreased in comparison between P deficiency and P supply under FL and LL. Leucocyanidin (from 1 to 5.92; *p* < 0.001) increased in FL but decreased (from 5.54 to 3.07; *p* < 0.01) under shading compared to other light intensities under P limitation. Cyanidin (from 2.64 to 4.1) increased significantly but cyanidin 3–glucoside (from 0.86 to 0.1) decreased significantly under P limitation compared to other light intensities.

### Overview of Targeted Metabolomics Analysis of Tea Plants Under P-Deficient and P-Sufficient Conditions

#### Young Shoots

**Figure 5:**
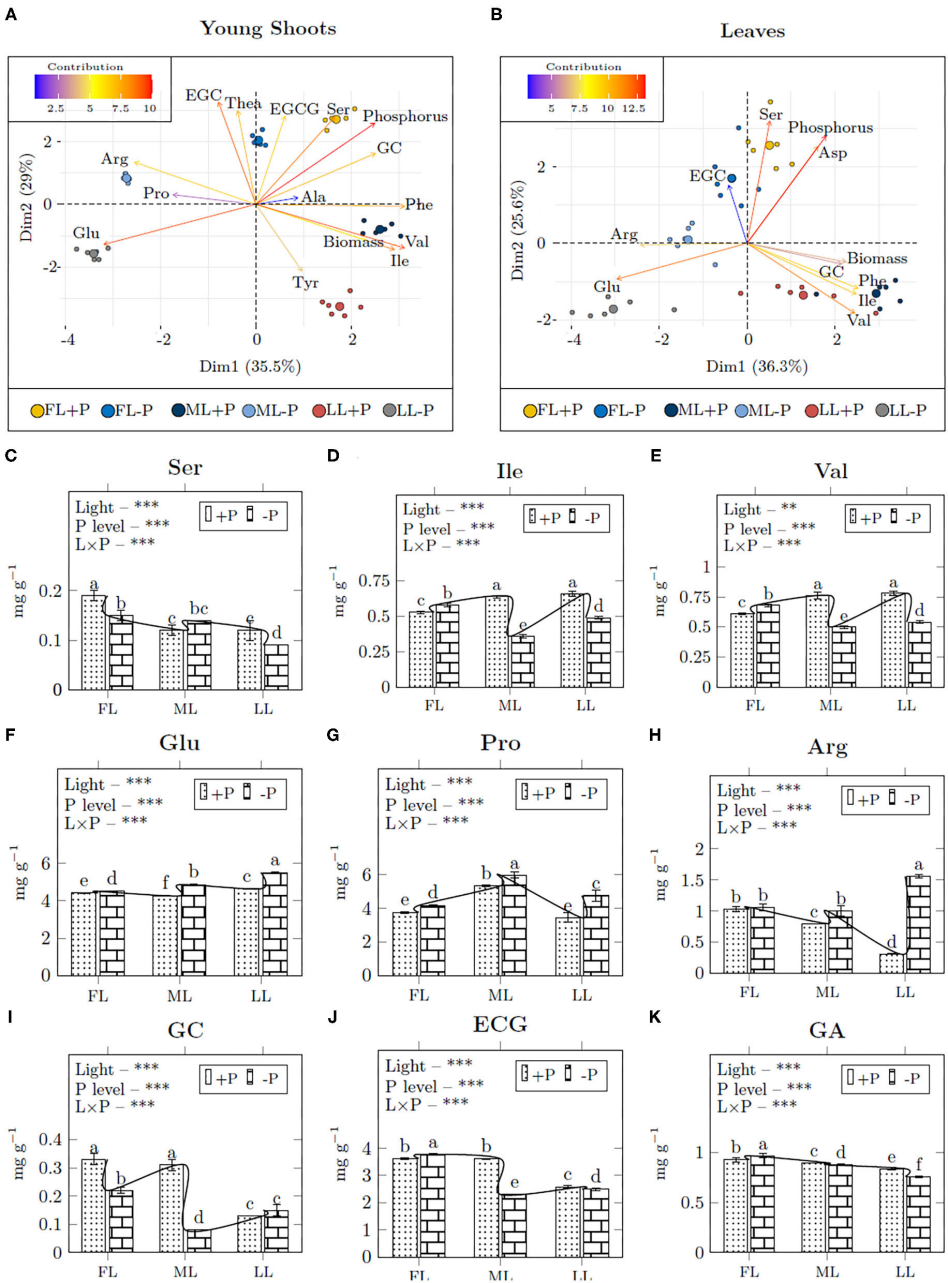
The bi–plot correlation of young shoots **(A)** and leaves **(B)** and the mean concentration of amino acids **(C–I)** and catechins **(J,K)** in young shoots. A single bi–plot for the dataset combines both samples and treatments to the principal components; Dim1, dimension 1, and Dim2, dimension 2. Asterisks indicate significant differences: ***is 0.001, **is 0.01, and *is 0.05, ns, non-significant differences, between light and P interaction.

The free amino acids and flavonoids contents were significantly altered by light and P treatment, and hence, were grouped based on light effect, P effect, or light-P interaction. The loading treatments from PCA directly link to correlation analysis for group separation (Figures 5A,B; Supplementary Table 5). In young shoots, P was grouped with Ser and GC that had high contribution on principal components (Figure 5A). The Ser content decreased in FL (0.79, –P/+P) and LL (0.75) in young shoots but it increased in ML (1.17) due to P limitation (Figure 5C). Similarly, shading decreased Ile (0.65) and Val (0.68) at P-deprivation (Figures 5C–E). Glu (1.17), Pro (1.22), and Arg (2.31) increased under shading due to P limitation (Figures 5F–H). GC (1.15) increased in LL, whereas ECG (0.77) and GA (0.94) decreased under shading due to P limitation (Figures 5I–K).

#### Leaves

In leaves, P was grouped with Ser and Asp with a high contribution on principal components (Figure 5B). P deficiency decreased the content of Ser in FL (0.67) and ML (0.53), however, there was a mild increase in LL (1.33) with the comparison between P deficiency and P supply under all light treatments (Supplementary Table 5). Ile (1.06) and Phe (1.13) increased in FL but shading decreased Ile (0.19), Val (0.5), and Phe (0.49) contents due to P limitation. Full light conditions decreased Asp (0.9) and Glu (0.87) under P limitation. Shading decreased Asp (0.45) but increased Glu (1.83) due to P limitation. Arg (3.74) increased in all light intensities at –P conditions. EGC (1.53) and GC (1.29) increased in FL due to P limitation. Shading increased EGC (1.61) but GC (0.58) was decreased in LL at –P conditions.

### Relative Expression of Genes in Response to P Limitation and Light Changes

**Figure 6:**
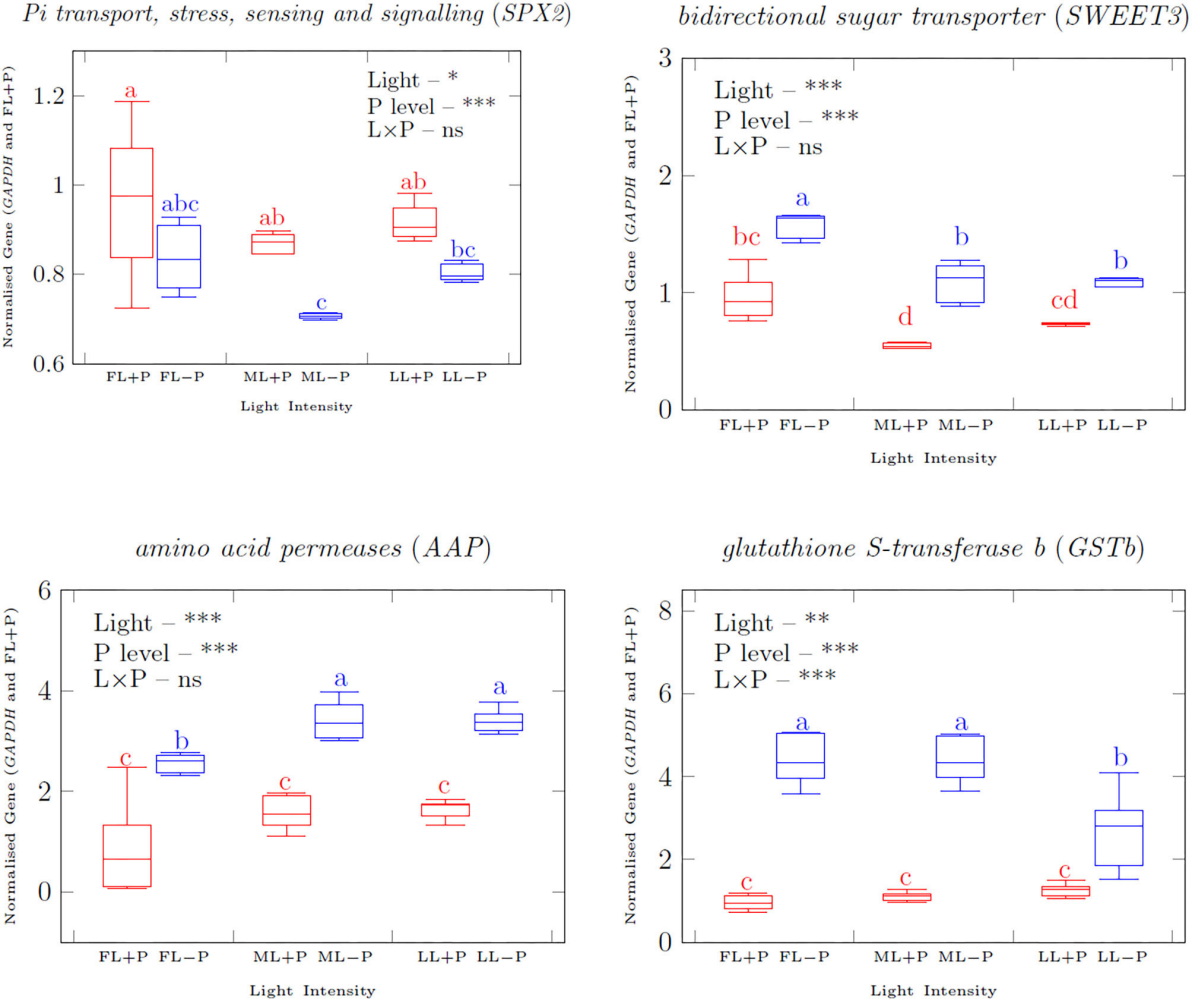
qRT–PCR analyzed (normalized with reference gene delta CT) transporter gene transcripts normalized relative expression in response to full light with +P. Asterisks indicate significant differences: ***is 0.001, ** is 0.01, and *is 0.05, ns, non-significant differences, between light and P interaction.

**Figure 7:**
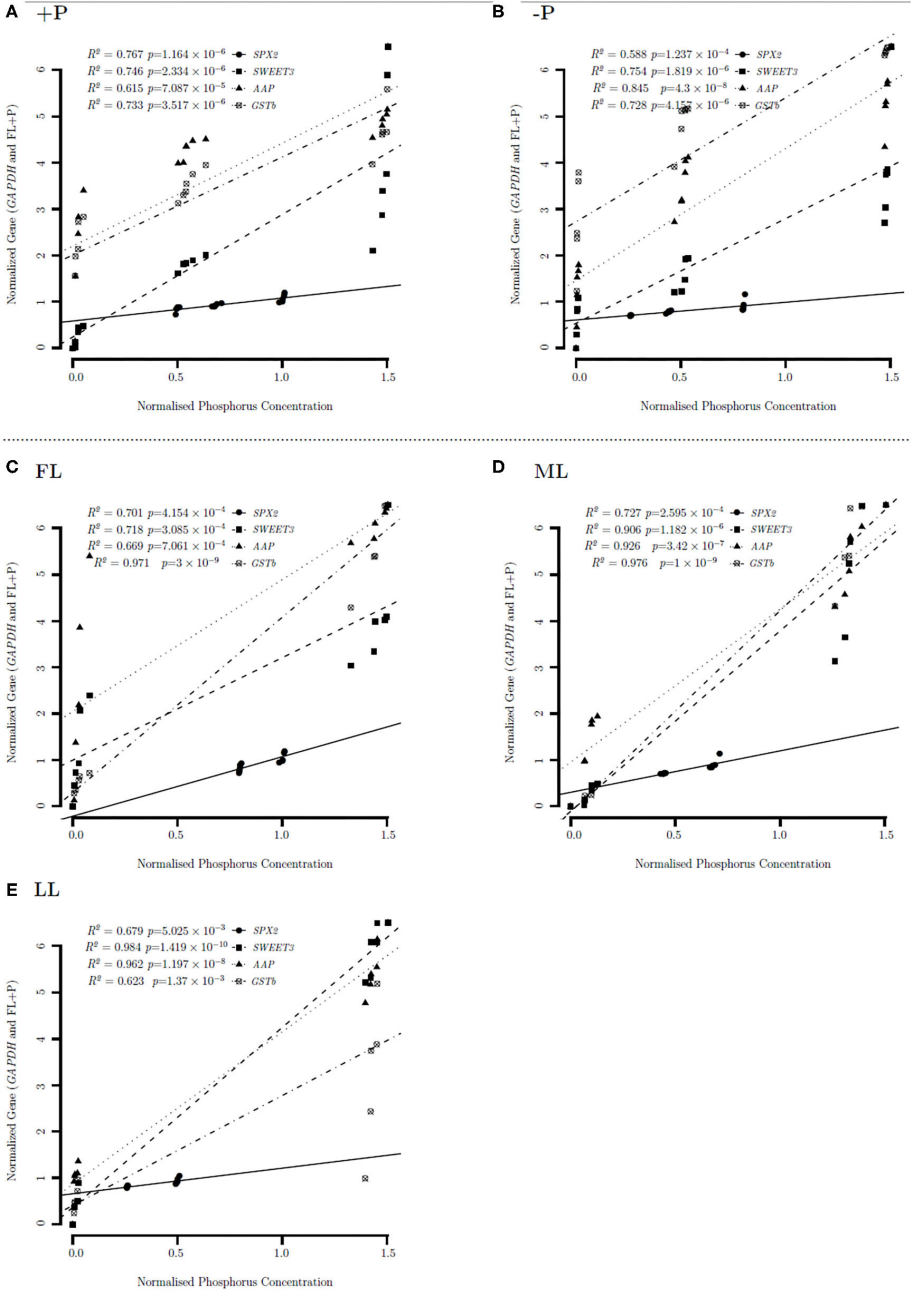
Linear regression correlation between gene expression under different P levels **(A,B)** and light regimes **(C–E)**. High P **(A)**, low P **(B)**, full light (FL) **(C)**, medium light (ML) **(D)**, and low light (LL) **(E)** correlation of gene expression under different P levels and light intensities of Pi transport, stress, and signaling *(SPX2)*, bidirectional sugar transporter 3 (*SWEET3*), amino acid permeases (*AAP*), and glutathione S-transferase b *(GSTb)* in young shoots.

The expression of the transcription factor *Pi transport, stress, sensing, and signaling (SPX2)* was upregulated by P supply under each light treatment (Figures 6, 7). Shading decreased *SPX2* expression level when P was limited. In addition, bidirectional sugar transporter *(SWEET3)* was upregulated due to P limitation while it was downregulated under shading conditions. P limitation increased the transcripts of the amino acid transporter gene amino acid permeases *(AAP)* under all the light treatments and it was also increased by shading treatments. The interactive effect of light and P on the expression levels of *SWEET 3* and *AAP* were not significant while it was significant on the transcript level of glutathione S-transferase b (*GSTb)*. There was an upregulation response of *GSTb*) by P limitation regardless of the light treatments. The expression of most metabolic genes investigated was upregulated in young shoots due to P limitation (Supplementary Figures 4–6) but a change in *arg* was not statistically significant. No significant interaction between light and P was found on the expression of ribose 5-phosphate isomerase A (*RPIA*), *arg*, and prolyl-4-hydroxylase (*P4H*). *Ribokinase synthase*, inositol-phosphate phosphatase *(IMPL1)*, xylose isomerase *(XylA)*, isocitrate dehydrogenase *(IDH)*, leucoanthocyanidin dioxygenase *(LDOX)*; however, anthocyanidin 3-O-glucosyltransferase (*UFGT*) was upregulated in LL due to P limitation. Shading upregulated *IDH* and *UFGT* metabolic genes. With increasing P concentration, the expression levels of *SWEET3, AAP*, and *GSTb* were highly induced.

### Heatmap Correlation Clustering of Metabolites and Gene Expression Under P-Deficient and P-Sufficient Conditions

**Figure 8:**
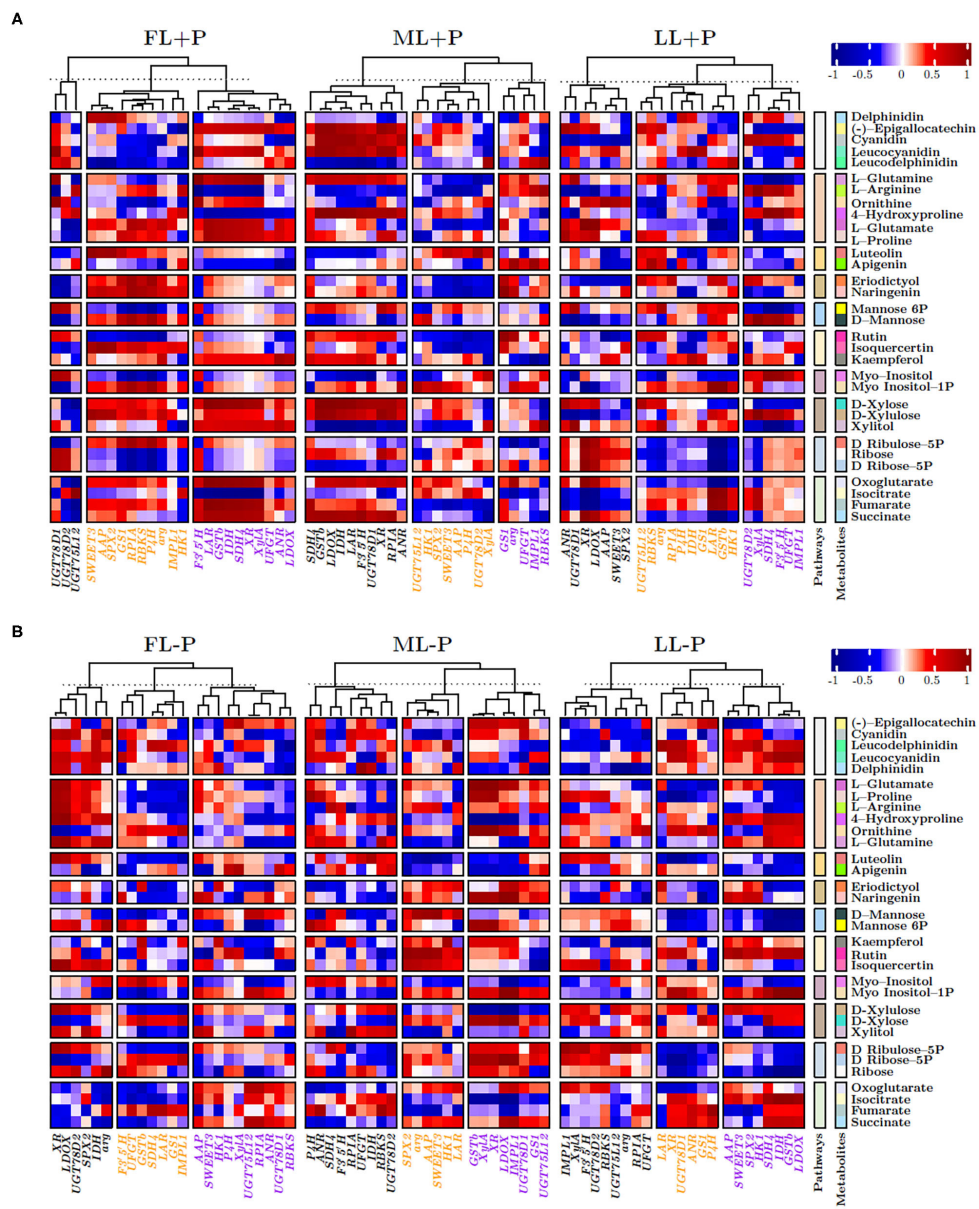
**(A,B)** Heatmap correlation between P and genes under different P levels and light intensities.

Figure 8A shows the heat map of correlation between biosynthetic pathway metabolites with the selected genes under P-sufficient conditions. Flavonol glycosides and flavones biosynthesis related genes, *UGT78D1, UGT78D2*, and *UGT75L12*, were clustered into the same hierarchal group to show similar expression patterns of correlations with all discussed pathways in FL at P-sufficient conditions (Figure 8A). In addition, *SWEET3, AAP, SPX2*, carbohydrate, and amino acid biosynthesis and/or related anaplerotic pathways related genes were clustered into the same hierarchal group to show similar expression levels of correlations in FL at P-sufficient conditions. Interestingly, *GSTb* was clustered with all secondary metabolic genes to show its distinct and similar expression of correlations in FL at P-sufficient conditions. Similarly, in ML at P-sufficient conditions, *SPX2, SWEET3*, and *AAP* were also clustered into the same hierarchal group and *GSTb* showed a different cluster pattern with metabolic genes. Likewise, in LL at P-sufficient conditions, *SPX2, SWEET3*, and *AAP* were also clustered into the same hierarchal group and *GSTb* showed a different hierarchal cluster pattern with metabolic genes. Notably under FL and P-deficient conditions, *SPX2* and *GSTb* formed a different cluster group while *SWEET3* and *AAP* were also clustered into the same hierarchal group to show a similar expression of correlations with metabolic genes (Figure 8B). Similarly, *SPX2, AAP*, and *SWEET3* were clustered into the same group with metabolic genes in ML and LL at P-deficient conditions.

### Overview of Metabolic Pathways and Involved Genes

**Figure 9:**
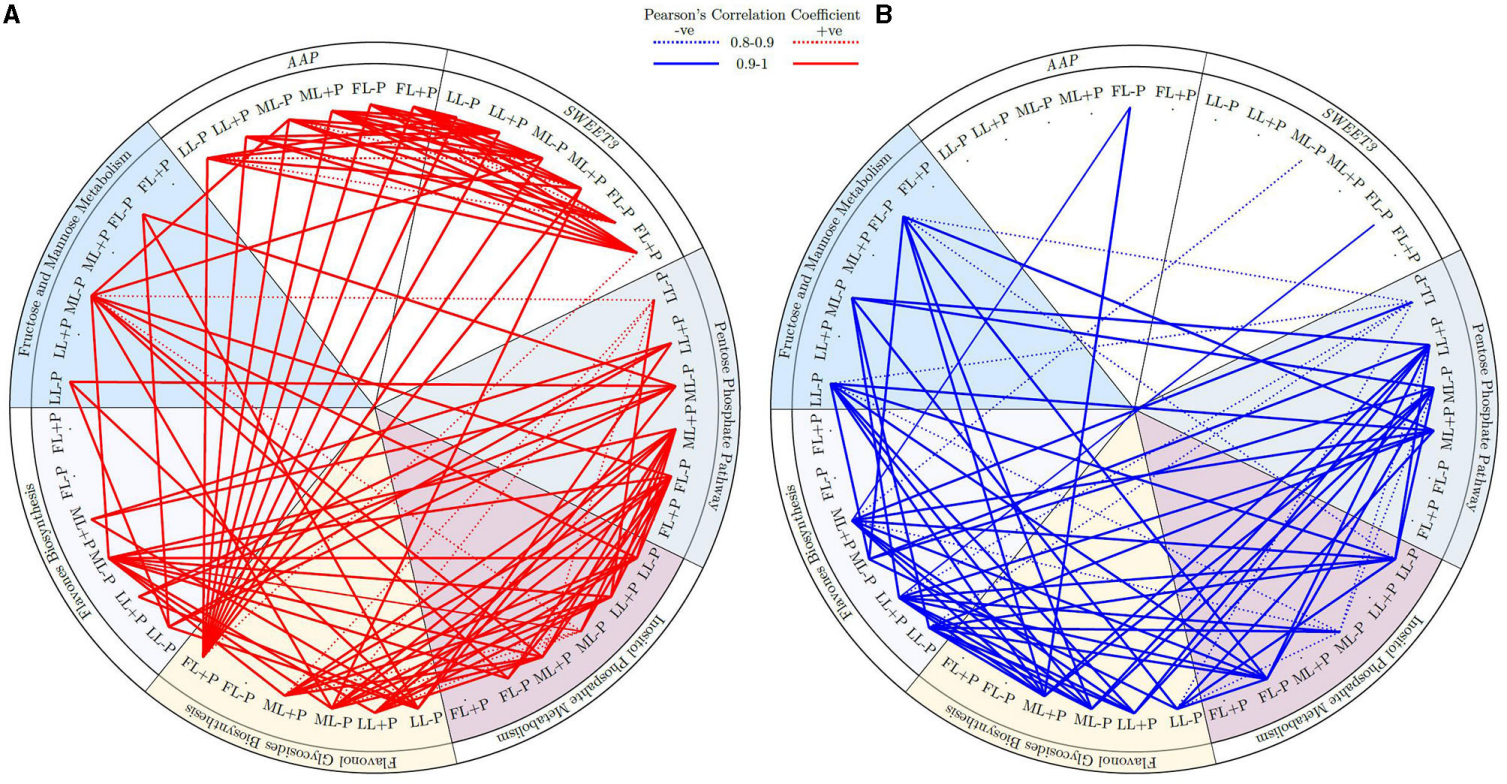
**(A,B)** Circular representation of the correlation between genes under different P levels and light intensities.

Amino acid permeases and *SWEET3* were positively correlated under different light and P regimes. Similarly, both *AAP* and *SWEET3* were positively correlated with flavonol glycosides biosynthesis in FL at P-sufficient conditions (Figure 9). Similarly, the cluster of fructose and mannose related metabolites in FL at P-deficient conditions was strongly correlated with pentose phosphate pathway at ML and flavonol glycosides, and flavones biosynthesis at LL due to P deficiency. In addition, under shading conditions, flavonol glycosides biosynthesis was positively correlated with inositol phosphate metabolism. There was a strong negative correlation between FL–P of *AAP* and ML+P of flavonol glycosides biosynthesis. Fructose and mannose metabolism (FMM) under P deficiency at FL conditions was negatively correlated with flavonol glycosides biosynthesis under ML conditions. FMM that was due to ML under P deficiency was negatively correlated with flavones biosynthesis under LL conditions. Flavones and flavonol glycosides metabolites under shading were negatively correlated with the cluster of pentose phosphate pathway metabolites under shading. Most of the clustered inositol phosphate metabolites that were due to P limitation were negatively correlated with flavones biosynthesis metabolites under shading conditions (Figure 9).

## Discussion

The interaction between light intensity and P has been widely reported in plants, such as soybean (Wang et al., 2020), maize (Zhou et al., 2020), and white lupin (Cheng et al., 2014). Previous studies have focused on the influence of light intensity on low-P induced root morphology and Pi acquisition. Nevertheless, how light intensity affects the P-related metabolic processes is yet to be understood, especially in the xylophyta tea plants which are sensitive to light changes. In the present study, the interaction between light intensity and P nutrition was identified in the shoot of tea plants and this interaction affected the carbohydrates, amino acids, flavonoids, and related anaplerotic pathways significantly.

In Fructose and Mannose metabolism, D–fructose, a monosaccharide, is transformed into mannose and further metabolized by HXK into mannose-6P. It has been reported that black tea contains high levels of Fructose (Stilo et al., 2020). The present study showed that either ML or P limitation at FL decreased D–fructose where it was metabolized into D–mannose in the young shoots (Figure 2). The decrease in D-fructose was further intensified under 80% shading combined with P limitation by upregulating the expression of *HXK*, which produces the substrate, mannose-6P. This is consistent with its role as a regulator for the crosstalk of sugars and Pi in Arabidopsis where *HXK*s were reported to be able to sense the sugar level and coordinate mineral uptake by the root (Lejay et al., 2008; Granot et al., 2014). In Inositol phosphate metabolism, Inositol-phosphate phosphatase 1 (EC 3.1.3.25) produces the substrate Myo–inositol which acts as a precursor with various roles in plant signal transduction and environmental responses (Gillaspy, 2011). In LL, both Myo-inositol-1P and Myo-inositol contents declined due to P deficiency (Figure 2). By contrast, the upregulated expression of *IMPL1* induced by P limitation decreased Myo-inositol-1P and increased Myo-inositol under full light conditions (Supplementary Figure 4; Figure 2), suggesting different signaling pathways in response to P deficiency at different light intensities. In addition, D-ribose-5P could result from phosphorylation of ribose by *RBKS* (EC 2.7.1.15) or synthesized from D–ribulose 5P by *RPIA* (EC 5.3.1.6). These processes play essential roles in carbohydrate anabolism and catabolism, for instance, in the pentose phosphate pathway that runs parallel to glycolysis (Kruger and von-Schaewen, 2003; Zhang et al., 2003). Under the FL and LL conditions, the induced expression of *RPIA* and *RBKS* by P limitation resulted in the accumulation of D-Ribose-5P (Figure 2; Supplementary Table 3, Supplementary Figure 4). Whereas D-ribose-5P decreased and ribose increased due to P limitation under ML although the expression of *RBKS* was induced, indicating differential regulation of the pathway in ML compared to FL and LL. In ML, ribose is a crucial source for NADPH generation due to P deficiency for reductive biosynthesis. Likewise, xylose isomerase (EC 5.3.1.5) catalyzes the interconversion of D–xylose to D–xylulose. The enzyme D–xylose reductase (EC 1.1.1.307) belongs to the aldose reductase family and catalyzes the reduction of D–xylose to the sugar alcohol xylitol (Quehenberger et al., 2019). The response of D-xylulose and L-xylulose is likely related to the opposite relation on carbon utilization. This causes L-xylulose partition on ethanol production which tends to the growth of young shoots despite shading and P limitation.

In the targeted metabolite analysis, the concentration of serine declined under shading in young shoots due to a reduction in the photosynthetic carbon assimilation (Timm et al., 2013). In contrast to FL and LL, an increase in serine by P limitation was detected in ML which was also reported previously in the P-deficient maize leaves (Schlüter et al., 2013). Shading treatment increased Ile and Val under P-sufficient conditions. This was a response that has also been reported previously for Arabidopsis (Gipson et al., 2017), presumably to preserve these essential amino acids for protein synthesis as an adaptive mechanism in young shoots. In general, most amino acids have been reported to present at higher levels in the shaded tea leaves (Yang et al., 2012). In the present study, phenylalanine was remarkably increased by half-light intensity compared with full light intensity when P was sufficient. However, the Phe concentration was reduced by further shading with 20% light intensity and also decreased by P deficiency. The reduction of Phe under low light and P deficiency indicates an irreversible effect on the physiology of the plant (Ku et al., 2010) due to its crucial roles in plant growth and defense against various types of stresses. It has been reported that asparagine transport contributes to nitrogen mobilization from source to sink organs (Oddy et al., 2020). Here, either P limitation or shading treatments reduced Asp concentration which may be associated with photorespiration and subsequently hindered nitrogen mobilization (Novitskaya et al., 2002; KC et al., 2018).

The enzymes of the TCA cycle act as a component in the electron transport chain where succinate can only be oxidized by succinate dehydrogenase (SDH). SDH activity depends on white light intensity and in our study, the expression of *SDH4* decreased with shading under P insufficient conditions (Supplementary Figure 5). This was a response that has also been reported on Arabidopsis (Popov et al., 2010). Furthermore, the induced expression of *SDH4* by P deficiency resulted in the decrease of succinate and increase of fumarate. In addition, the reduced expression of *SDH4* by shading caused the accumulation of succinate and decrease of fumarate, suggesting the transcriptional regulation on *SDH4* by light and P. The enzyme GS1, located in the cytosol, contributes to metabolic systems *via* the regulation of ammonium assimilation (Kusano et al., 2020). The decrease in substrate L–glutamine and reduction in expression of *GS1* negatively affect the amino acid homeostasis and plastid development in young shoots due to shading and P limitation. The arginine and proline metabolism plays critical roles in plant development and stress resistance that regulates polyamines independently (Majumdar et al., 2016). The response of *P4H* expression was not consistent with the changes of proline and 4-hydroxyproline due to the diverse substrates for P4H (Gorres and Raines, 2010). Arg catabolism serves to mobilize the stored nitrogen and fine-tuning the developmental and defense mechanisms against stress (Winter et al., 2015). Thus, it was assumed that the increase of Arg due to P deficiency under LL may regulate physiological and developmental processes, as well as responses to abiotic stress.

Several studies have shown that light promotes catechin synthesis and shading was effective for inhibition of the flavonoid biosynthesis (Wang et al., 2012; Li et al., 2018). Similarly, our data shows decreased catechins (ECG, EGC, and EGCG) under ML when P was sufficient. A compromised 3–*O*–glycosylation led to the repression of flavonol biosynthesis along with the inhibition of flavanol biosynthetic genes (Yin et al., 2012). The 3–*O*–glycosylation of flavanols is catalyzed by the UGT78D family, with UGT78D1 using UDP–rhamnose, and UGT78D2 using UDP–glucose (Yonekura–Sakakibara et al., 2008). In flavonol glycosides biosynthesis, the substrate isoquercertin was decreased due to P limitation combined with shading, indicating that UDP–glucose was highly consumed while UDP–rhamnose was highly utilized to form the substrate rutin when exposed to medium light during P deficiency in young shoots. The enzyme F3′5′H hydroxylates the 3′ and 5′ positions of dihydroflavonols (Tanaka and Brugliera, 2013; Vikhorev et al., 2019). The decrease in eriodictyol and increase in F3′5′H compete for substrate naringenin during P deficiency in ML. Leucoanthocyanidin reductase (LARs) has been reported to promote the biosynthesis of catechin monomers and inhibit their polymerization (Wang et al., 2018). In contrast, our data show that catechin monomers and polymers were increased by upregulating the expression of *LAR* (Wang et al., 2018). This could be due to the differences between the tea plants and tobacco (*Nicotiana tabacum*) which were domesticated for distinct purposes. In addition, the increase in Delphinidin−3–glucoside may have been supported by *UFGT* which was upregulated due to P limitation and shading. Similar results have been reported previously (KC et al., 2018). The enzyme LDOX is involved in anthocyanidin and anthocyanidins biosynthesis by catalyzing the oxidation of leucoanthocyanidins to cyanidins. Our results agree with a previous report where LDOX and its synthesized cyanidins increased due to P limitation. Furthermore, Cyanidin 3–glucoside, involved in energy metabolism and synthesized by anthocyanidin reductase *(ANR)*, was increased by shading.

Sensing the changes in internal nutrients is essential for plants to reprogram and adapt to fluctuating environments. For instance, the cellular P levels activate the central regulator for P starvation responses and P homeostasis (Wang et al., 2014). In rice, *SPX2* functions as a key component in the P-sensing mechanism to control the central regulator of P starvation-responsive genes. Under high cellular P, *OsSPX2* interacts with the central regulator *OsPHR2* and inhibits *PHR2* from binding to the motif of phosphate starvation inducible (PSI) genes. However, under low cellular P conditions, *OsSPX2* has a low affinity to *PHR2* and results in the upregulation of PSI genes, including *OsSPX2*. In tea plants, most of the metabolic genes detected in the present study were increased by P starvation; however, the transcripts of *SPX2* were decreased by P limitation, suggesting different sensing and regulating networks in tea and rice plants. It has been reported that *CsSWEET3* was downregulated under cold stress and the overexpression of the *SWEET* gene in Arabidopsis was unable to accumulate Fructose (Yue et al., 2015). This study shows that P limitation increased the transcript level of *SWEET3*; however, it decreased due to light reduction. D–fructose was increased by P limitation under ML and decreased by shading when P was sufficient. Environmental signals, such as nutrient availability and light influence the expression of *AAP* (amino acid permeases; Ortiz–Lopez et al., 2000; Yao et al., 2020). It has been reported that *AAP1* functions in the acquisition of glutamate if present in the soil at ecologically relevant concentrations (Lee et al., 2007). In this study, the linear regression and concentration measurement revealed that the increased concentration of L–glutamate in young shoots was positively correlated with the expression of *AAP* which was upregulated by P limitation combined with shading. In addition, a higher concentration of L–glutamate was observed in young shoots than in mature leaves, suggesting the mobilization of Glu from source to sink. A previous study showed that *GSTb* had a weak affinity for epicatechin which was decreased under P limitation but its gallates were increased in young shoots (KC et al., 2018; Liu et al., 2019). This study agrees with the previous findings that epicatechin gallates were increased in young shoots due to P deprivation.

## Conclusion

In conclusion, the two environmental factors light intensity and P supply interacted at the transcriptional and metabolic levels. The genes and metabolites involved in amino acids, carbohydrates, and flavonoid pathways were responsive to P-deficiency and shading. Different light intensities modulated some of the metabolites (e.g., Thea, EGC) differentially. Our data shows a comprehensive analysis of tea plants in response to changing light intensities coupled with P limitation. This information contributes to agricultural management to improve the quality of tea and maintain the yield.

## Data Availability Statement

The original contributions presented in the study are included in the article/Supplementary Material, further inquiries can be directed to the corresponding author/s.

## Author Contributions

JR conceived and designed the research. SK, LL, ML, and QZ performed the experiments and data interpretation. SK and LL wrote and revised the manuscript. All authors read and approved the manuscript.

## Funding

This work was financially supported by Yunnan provincial special fund for construction of science and technology innovation base (202102AE090038), Chinese Academy of Agricultural Sciences through Agricultural Sciences Innovation Project (CAAS-ASTIP-2020-TRICAAS), China Agriculture Research System of MOF and MARA (CARS 19), Special fund for scientific research of Tea Research Institute of Chinese Academy of Agricultural Sciences (grant no.1610212021001), and Zhejiang Provincial Natural Science Foundation of China (LY21C150005).

## Conflict of Interest

The authors declare that the research was conducted in the absence of any commercial or financial relationships that could be construed as a potential conflict of interest.

## Publisher's Note

All claims expressed in this article are solely those of the authors and do not necessarily represent those of their affiliated organizations, or those of the publisher, the editors and the reviewers. Any product that may be evaluated in this article, or claim that may be made by its manufacturer, is not guaranteed or endorsed by the publisher.

## Glossary

*HXK1, hexokinase 1*; *RPIA, ribose 5-phosphate isomerase A*; *GS1, glutamate synthetase 1*; *P4H, prolyl 4-hydroxylase*; Thea, Theanine; GC × GC–TOF/MS, Two-dimensional Gas Chromatography coupled to Time-of-Flight Mass Spectrometry; UPLC–Q–TOF/MS, Ultra-Performance Liquid Chromatography-Quadrupole-Time of Flight Mass Spectrometry; ICP–AES, Inductively Coupled Plasma-Atomic Emission Spectroscopy; qRT–PCR, quantitative Real Time-Polymerase Chain Reaction; GAPDH, glyceraldehyde 3-phosphate dehydrogenase; PCA, Principal Component Analysis; PLS–DA, Partial Least Squares-Discriminant Analysis; Glu, Glutamate; Pro, Proline; Arg, Arginine; EGC, Epigallocatechin; GC, Gallocatechin; GCG, Gallocatechin gallate; GA, Gallic acid; *SPX2, Pi transport, stress, sensing, and signaling*; *SWEET3, bidirectional sugar transporter*; *AAP, amino acid permeases*; *GSTb, glutathione S-transferase b; RBKS, ribokinase synthase; IMPL1, inositol-phosphate phosphatase 1; XylA, xylose isomerase; IDH, isocitrate dehydrogenase; LDOX, leucoanthocyanidin dioxygenase; UFGT, anthocyanidin 3-O-glucosyltransferase; UGT8D1, flavonol-3-O-glucoside L-rhamnosyltransferase; UGT78D2, flavonol-3-O-glycosyltransferase; UGT75L12, glucosyltransferases*; FMM, Fructose and Mannose Metabolism; TCA, Tricarboxylic acid cycle; SDH, Succinate dehydrogenase; *F3'5'H, flavonoid 3',5'-hydroxylase*; *ANR, anthocyanidin reductase*; PHR, phosphate starvation response.
