# Supplementary material for: Light Intensity Modulates the Effect of Phosphate Limitation on Carbohydrates, Amino Acids, and Catechins in Tea Plants (Camellia sinensis L.)
Source: Front Plant Sci. 2021 Oct 8;12:743781. doi: 10.3389/fpls.2021.743781 (PMC8532574; doi:10.3389/fpls.2021.743781)
Supplement: Supplementary file 1 [file Data_Sheet_1.pdf]

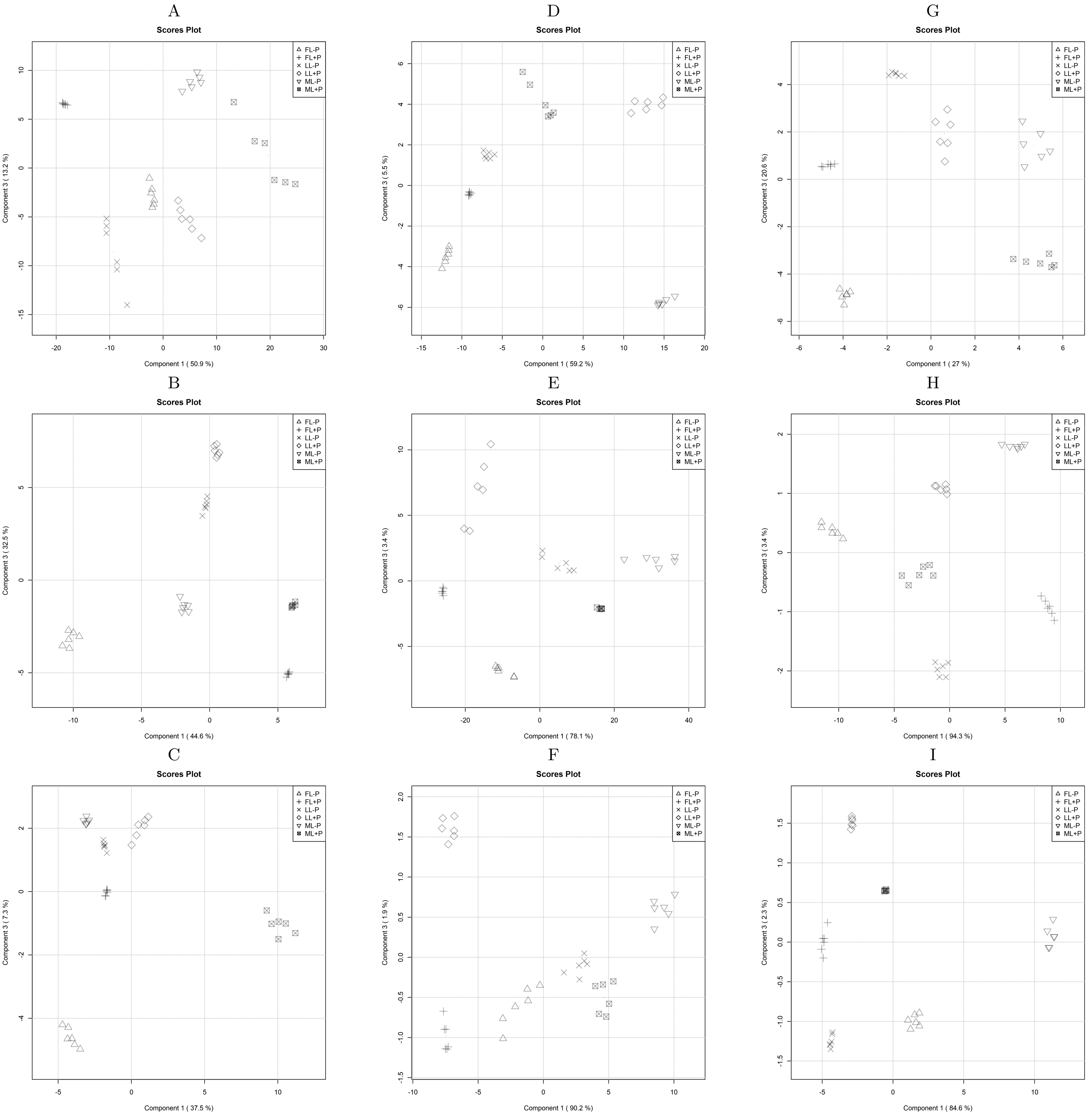

Figure S1. PCA analysis of metabolites in response to changes in light intensity and P availability. The cluster effect on abundance of carbohydrates (A-C), amino acids (D-F) and flavonoids (G-I) or related anaplerotic pathway metabolites in young shoots (A,D,G), leaves (B,E,H) and roots (C,F,I).

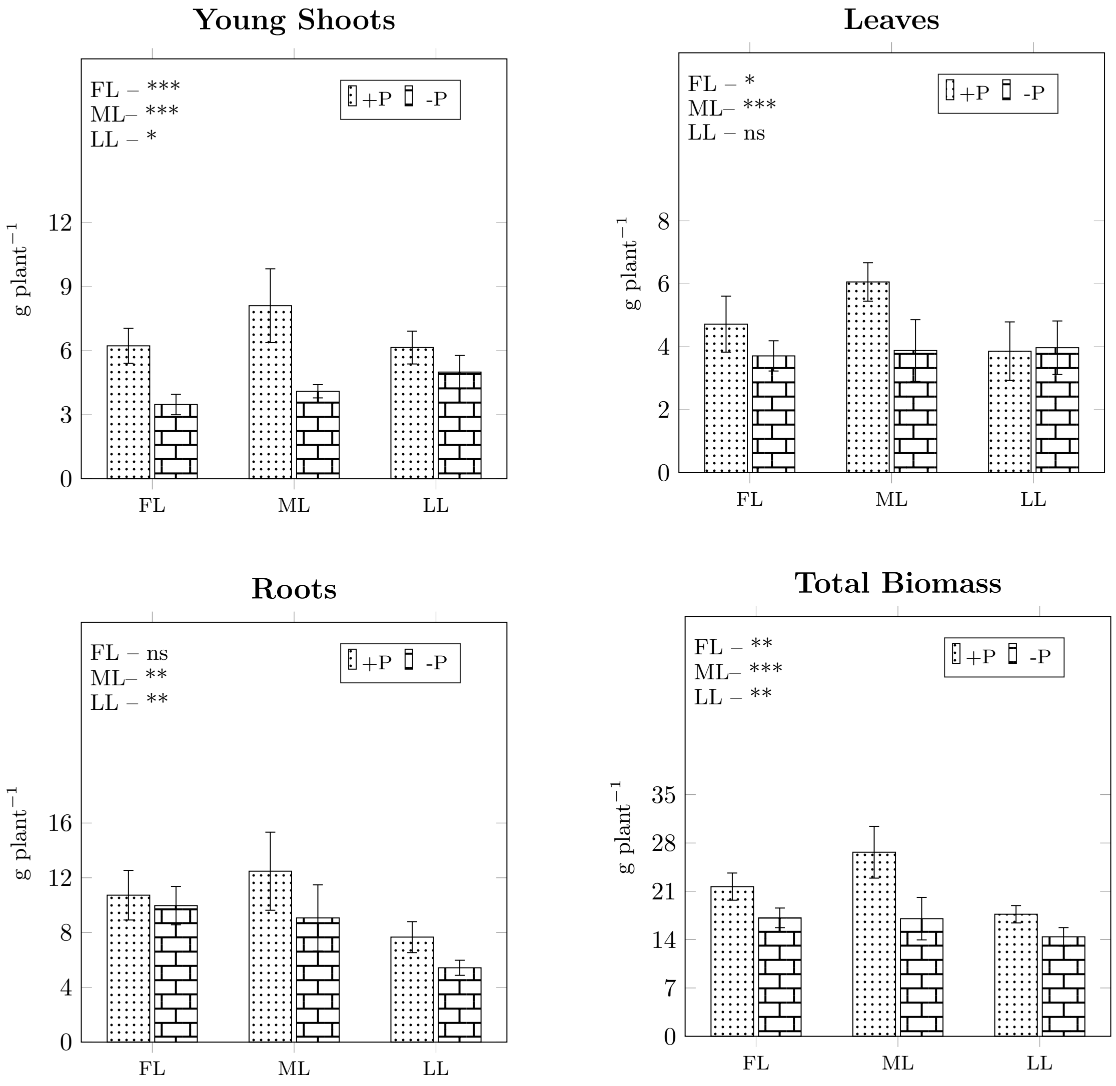

Figure S2. The error bar graph of mean of biomass (g plant^{-1}) exposed under FL, ML and LL conditions.

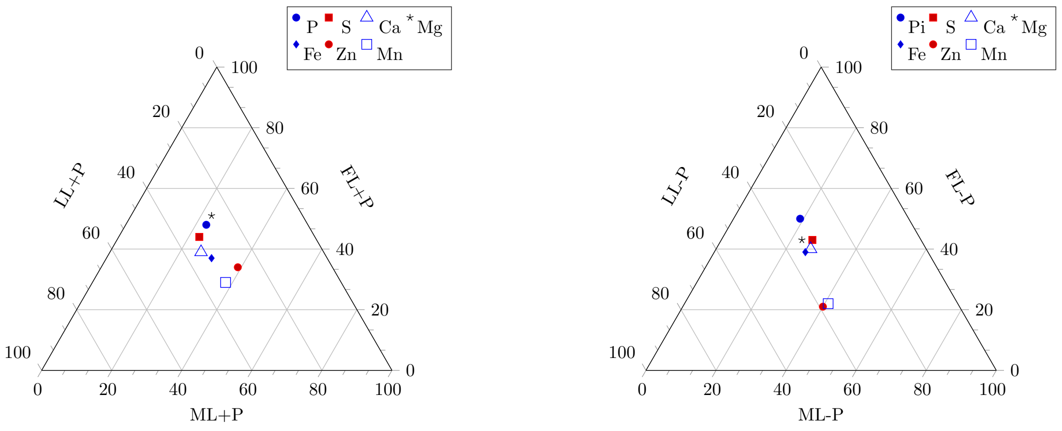

Figure S3. Ternary graph of plant nutrition in young shoots exposed to P sufficient (A) and P deficient (B) conditions..

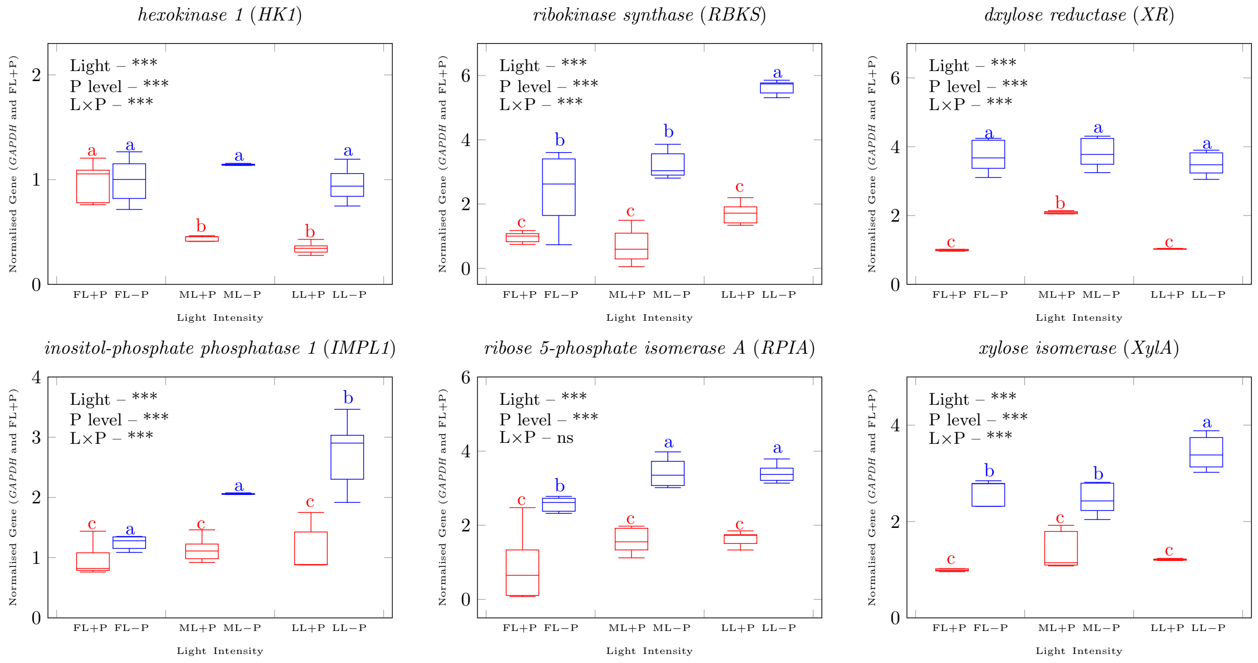

Figure S4. Quantification of the expression of different carbohydrates realted genes by qRT–PCR analyzed as normalized relative expression with gene and in response to changes in full light intensity and P availability..

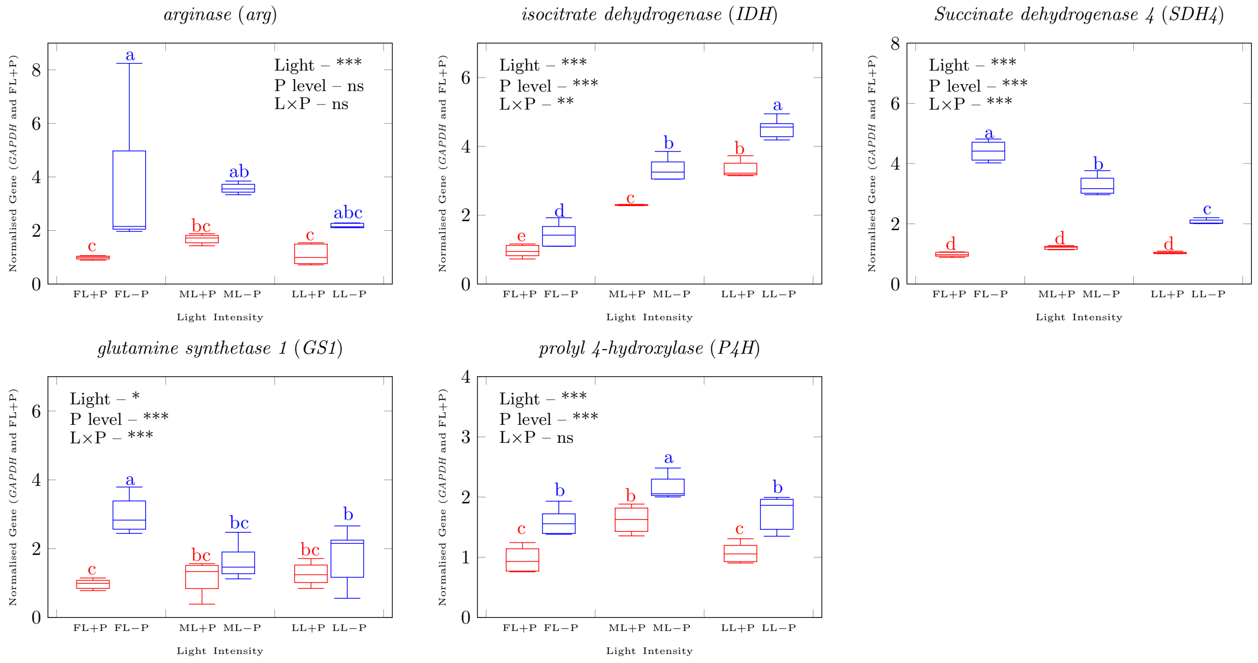

Figure S5. Quantification of the expression of different amino acids related genes by qRT–PCR analyzed as normalized relative expression with related gene and in response to changes in full light intensity and P availability.

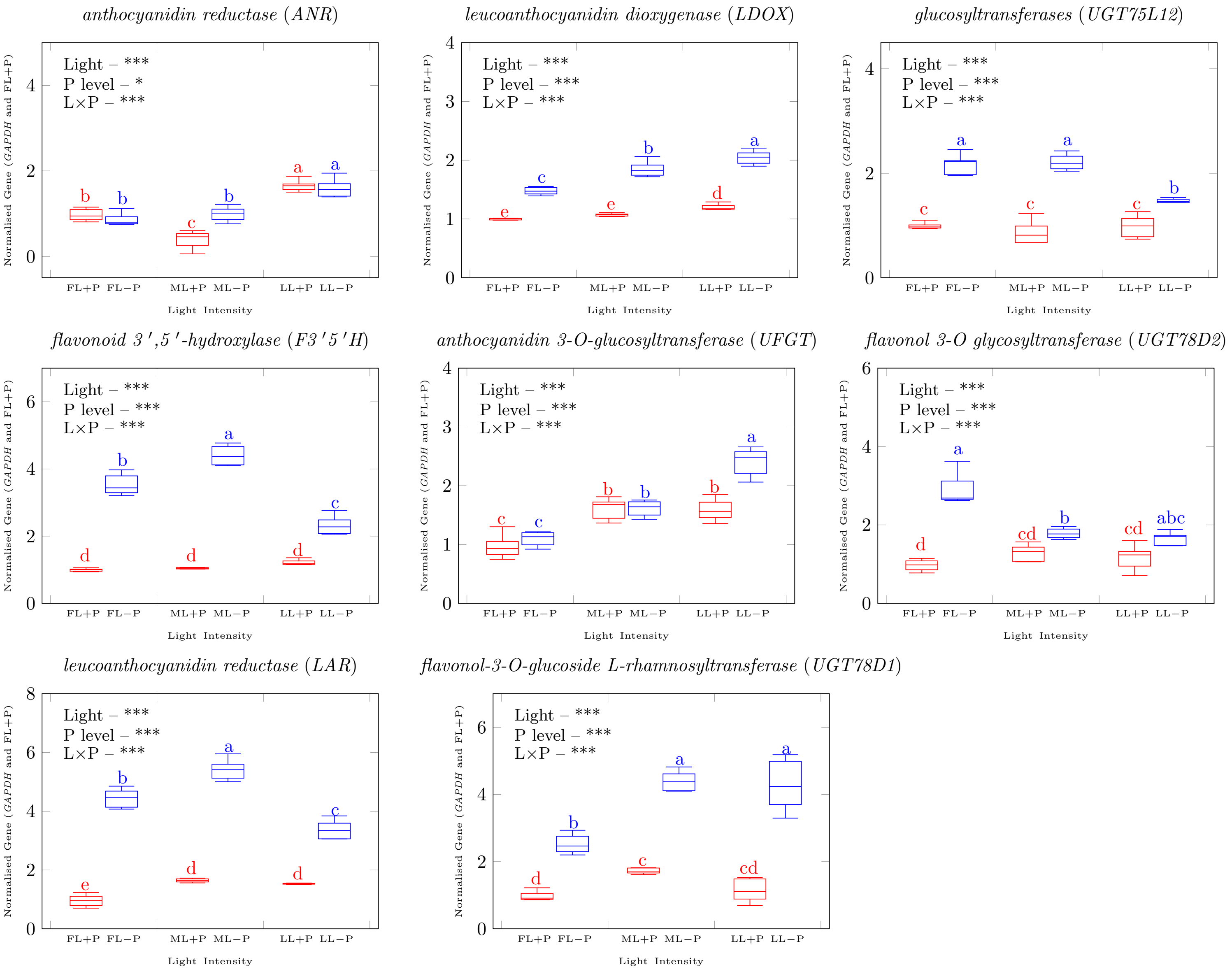

Figure S6. Quantification of the expression of different flavonoid related genes by qRT–PCR analyzed as normalized relative expression with gene and in response to changes in full light intensity and P availability.
