# Supplementary material for: Light Intensity Modulates the Effect of Phosphate Limitation on Carbohydrates, Amino Acids, and Catechins in Tea Plants (Camellia sinensis L.)
Source: Front Plant Sci. 2021 Oct 8;12:743781. doi: 10.3389/fpls.2021.743781 (PMC8532574; doi:10.3389/fpls.2021.743781)
Supplement: Supplementary file 2 [file Data_Sheet_2.pdf]

Table S1. Primer sequences for Fengqing cultivar for qRT-PCR analysis.

Gene	Gene Identifier	Forward primer (5'>3')	Reverse primer (5'>3')
SPX2 (*Pi transport, stress, sensing and signalling*)	CL58020Contig1	GGTGGCAACGAGAACTCACT	AACCGTCAACTTCTGCCCAT
SWEET3 (*bidirectional sugar transporter*)	CL9048Contig1	GGAGATCGGCTTCACCTGTC	ACAGGCAAGCCATACCAAGTA
AAP (*amino acid permeases*)	CL91534Contig1	CTGTGAGGGAAGGTACAGCC	GAGCAGGCAAAACAAACCCT
GSTb (*glutathione S-transferase b*)	CL79160Contig1	AGTGGTTCTTGTGAGTTTCTGGA	ACCCTTCTCTTCAAGGGCAAT

Table S2. Primer sequences for the gene transcripts of Fengqing cultivar of selected pathways for qRT-PCR analysis.

Pathway	Gene, reaction and Enzymatic activity	Gene Identifier	Forward primer (5'>3')	Reverse primer (5'>3')
Fructose and Mannose metabolism	*hexokinase 1* (2.7.1.1, *HK1*)	CL42645Contig1	CCCGTGTCGCAACTATGTCA	CGGCTCTCTTCGGTGAAACT
Inositol phosphate metabolism	*inositol-phosphate phosphatase 1* (3.1.3.25, *IMPL1*)	CL37584Contig1	TCGATTTCGGGTGTCTTGTAGG	TGATGACATGGTCGCATCCAA
Pentose phosphate pathway	*ribose 5-phosphate isomerase A* (5.3.1.6, *R5PIA*)	CL76822Contig1	GACTCGGAGTGATGGCAAGT	AATGGTAGGCACATGGAGGC
	ribokinase synthase (2.7.1.15, *RBKS*)	CL66999Contig1	ACAACCCCACAACCCTCAAG	AGACTGGCCAAACACTTGCT
Pentose and Glucuronate Interconversions	*xylose isomerase* (5.3.1.5, *XylA*)	CL2313Contig1	GTCGGATTGGCTTCGCTTTG	AGGATTAGTAGCAGCACCGC
	D-xylose reductase (1.1.1.15, *XR*)	CL23910Contig1	CCGATAGACTTCACCAGCCC	TCACGTTCCGATTGAAGCGA
Alanine, Asparate and Glutamate Metabolism	*glutamine synthetase 1* (6.3.1.2, *GS1*)	CL66758Contig1	TCCAAGCTTTTCGATGGCCT	GTGGAGAGCCAATCCCAACA
Kerb's cycle	*isocitrate dehydrogenase* (1.1.1.42, *IDH*)	CL31117Contig1	TACGCGTGCCTAATCAGGAG	AGGTGTTGAACCCAGTGGTG
	Succinate dehydrogenase 4 (1.3.5.1, *SDH4*)	CL33432Contig1	CCTACGACAACCGCATCGT	CCCCACATTCGAAGACCCTT
Arginine and Proline biosynthesis	*prolyl 4-hydroxylase* (1.14.11.2, *P4H*)	CL27105Contig1	CGTCGCCTTTCTTCGGTTTC	TCTGATGTGACCAAAGGCGG
	arginase (3.5.3.1, *arg*)	CL40328Contig1	CCGCTCTCTTCATCAGCTCC	ACCTCCTAAAGCACGCACAA
Flavonol Glycosides Biosynthesis	*flavonol 3-O glycosyltransferase* (2.4.1.91, *UGT78D2*)	CL89596Contig1	CAGGGATGGAACATGTCGCT	AAATTACCCACCGCCGAGTT
	flavonol-3-O-glucoside L-rhamnosyltransferase (2.4.1.159, *UGT78D1*)	CL25776Contig1	AACCATGGGTCCCAATAGCG	ACAGAATAACTCGCCGCACA
Flavanones Biosynthesis	*flavonoid 3',5'-hydroxylase* (1.14.14.81, *F3' 5' H)*	CL54192Contig1	CATCTCCGCTAGTGCCCATT	ATGATGAGAGGCTCAGCGTG
Anthocyanidins Biosynthesis	*leucoanthocyanidin reductase* (1.17.1.3, *LAR*)	CL77394Contig1	CTGCAGCAAGGAGGTCATCT	TCAGGGGTGCCCTACACTTA
	*anthocyanidin 3-*O*-glucosyltransferase* (2.4.1.115, *UFGT*)	CL89161Contig1	CATAGGCTCCCGAAGCAGAG	GAATCACCGCCAGTTTACGC
Flavones Biosynthesis	*glucosyltransferases* (2.1.4.81, *UGT75L12*)	CL90073Contig1	AGTTCTTTCGCACCCGTCAT	TCTTCCACCAGCTTCGCATT
Anthocyanin and Anthocyanidins Biosynthesis	*leucoanthocyanidin dioxygenase* (1.4.11.19, *LDOX*)	CL59191Contig1	CAACCGGCTTCCATACCCTT	TTGGCGATGACAGTGCTCTT
	anthocyanidin reductase (1.3.1.77, *ANR*)	CL2560Contig1	ACTTGTGTTGTTGGCGATCC	ACAAGGCTAGGGCTAAAGCA

Table S3. Light intensity and P interaction effect on Primary metabolites and/or related anaplerotic pathway metabolites to carbohydrates and amino acids of Fengqing cultivar measured by GC×GC-TOF/MS analysis (Data normalise to 1 based on treatment (FL+P).

					Light Intensity			Significance						
Metabolites	Mass	CAS	Organ	P level	Full Light (FL)	Medium Light (ML)	Low Light (LL)	Light (L)	P level (P)	L×P	FL	ML	LL	FL×(ML+LL)
D-Fructose	204	57-48-7	YS	+P	1 ± 0.17ab	0.65 ± 0.3b	0.41 ± 0.03b	**	**	ns	-	-	-	-
				-P	0.69 ± 0.05ab	1.35 ± 0.86a	0.45 ± 0.11b							
			Leaves	+P	1 ± 0.05b	1.25 ± 0.02a	0.73 ± 0.02c	***	***	**	-	-	-	-
				-P	1.29 ± 0.01a	1.31 ± 0.21a	0.86 ± 0bc							
			Roots	+P	1 ± 0.03d	1.75 ± 0.02a	1.15 ± 0.02c	***	***	***	-	-	-	-
				-P	1.43 ± 0.14b	0.62 ± 0.03e	0.41 ± 0.13f							
D-Mannose	103	3458-28-4; 31103-86-3	YS	+P	1 ± 0.11bc	1.75 ± 0.96bc	0.3 ± 0.02bc	***	***	***	***	**	***	***
				-P	13.4 ± 3a	0.03 ± 0.02c	2.38 ± 0.02b							
			Leaves	+P	1 ± 0.05a	0.34 ± 0.1c	0.13 ± 0.01d	***	**	***	-	-	-	-
				-P	0.92 ± 0.16a	0.11 ± 0.01d	0.75 ± 0.06b							
			Roots	+P	1 ± 0.07c	0.64 ± 0.08d	0.56 ± 0.03d	***	***	***	***	**	***	***
				-P	4.64 ± 0.19a	0.5 ± 0.03d	1.92 ± 0.02b							
Mannose 6P	204	3672-15-9	YS	+P	1 ± 0.04a	0.63 ± 0.01b	0.97 ± 0.07a	***	***	***	***	***	***	*
				-P	0.61 ± 0.03bc	0.55 ± 0.02c	0.28 ± 0.02d							
			Leaves	+P	1 ± 0c	1.22 ± 0.01ab	1.3 ± 0.14a	***	***	***	-	-	-	-
				-P	1.19 ± 0.02b	0.82 ± 0d	0.69 ± 0.02e							
			Roots	+P	1 ± 0.08c	4.46 ± 0.36a	4.99 ± 0.7a	***	***	***	***	***	***	*
				-P	1.74 ± 0.06b	1.1 ± 0.09c	0.62 ± 0.03c							
Myo Inositol-1P	259	15421-51-9	YS	+P	1 ± 0.03ab	0.31 ± 0.2c	1.11 ± 0.05a	***	***	***	**	ns	***	**
				-P	0.76 ± 0.13b	0.42 ± 0.25c	0.44 ± 0.18c							
			Leaves	+P	1 ± 0.01e	1.14 ± 0.01de	3.78 ± 0.18a	***	**	***	-	-	-	-
				-P	3.16 ± 0.18b	1.54 ± 0.21d	2.07 ± 0.57c							
			Roots	+P	1 ± 0.05b	0.56 ± 0.01d	2.18 ± 0.18a	***	***	***	***	***	***	ns
				-P	0.76 ± 0.09c	0.63 ± 0.01cd	0.6 ± 0.01d							
Myo Inositol	44	87-89-8	YS	+P	1 ± 0.04c	1.08 ± 0.06c	2.76 ± 0.31b	***	***	***	***	ns	***	***
				-P	8.81 ± 1.98a	0.96 ± 0.13c	1.01 ± 0.03c							
			Leaves	+P	1 ± 0.03c	0.97 ± 0.01cd	1.24 ± 0.06b	***	*	***	-	-	-	-
				-P	1.52 ± 0.02a	0.66 ± 0.03e	0.85 ± 0.16d							
			Roots	+P	1 ± 0.01e	1.69 ± 0.03c	1.88 ± 0.03b	***	***	***	***	***	***	**
				-P	3.7 ± 0.18a	1.04 ± 0.05e	1.2 ± 0.02d							
Gluconate	174	526-95-4	YS	+P	1 ± 0.12d	7.85 ± 0.18b	7.82 ± 0.46b	***	***	ns	***	***	***	ns
				-P	8.57 ± 0.55a	1.84 ± 0.11c	1.79 ± 0.04c							
			Leaves	+P	1 ± 0.01e	1.17 ± 0.07e	14.85 ± 0.03a	***	***	***	-	-	-	-
				-P	7.41 ± 0.46c	5.31 ± 0.27d	10 ± 0.01b							
			Roots	+P	1 ± 0.09a	0.94 ± 0.65a	0.15 ± 0.01b	***	***	**	-	-	-	-
				-P	0.11 ± 0b	0.77 ± 0.1a	0.12 ± 0.01b							
Glu6P	387	3671-99-6	YS	+P	1 ± 0.21de	2.92 ± 0.35b	1.49 ± 0.32cd	***	***	***	-	-	-	-
				-P	1.89 ± 0.11c	5.72 ± 0.95a	0.32 ± 0.1e							
			Leaves	+P	1 ± 0b	0.73 ± 0.06c	0.73 ± 0.08c	***	***	***	-	-	-	-
				-P	1.16 ± 0.01a	0.24 ± 0.09d	0.82 ± 0c							
			Roots	+P	1 ± 0.05ab	1.13 ± 0.05a	0.88 ± 0.26b	***	***	***	-	-	-	-
				-P	0.68 ± 0.02c	0.11 ± 0.03d	0.11 ± 0.02d							
D-Ribulose 5P	243	551-85-9	YS	+P	1 ± 0.11d	0.33 ± 0.08e	2.14 ± 0.08b	***	***	***	-	-	-	-
				-P	1.3 ± 0.07c	1.15 ± 0.12cd	2.75 ± 0.07a							
			Leaves	+P	1 ± 0.07a	0.16 ± 0.01e	0.55 ± 0bc	***	***	***	-	-	-	-
				-P	0.51 ± 0.03c	0.33 ± 0.02d	0.62 ± 0.07b							
			Roots	+P	1 ± 0.05d	0.79 ± 0.02e	1.36 ± 0.03b	***	***	***	***	ns	***	***
				-P	1.18 ± 0.02c	0.8 ± 0.03e	3.18 ± 0.05a							
D Ribose-5P	39	4300-28-1	YS	+P	1 ± 0.01b	0.73 ± 0.03c	0.15 ± 0.08e	***	***	***	-	-	-	-
				-P	1.27 ± 0.11a	0.42 ± 0.02d	1.32 ± 0.17a							
			Leaves	+P	1 ± 0b	0.54 ± 0.09c	0.18 ± 0d	***	***	***	-	-	-	-
				-P	0.96 ± 0.01b	0.28 ± 0.01d	1.31 ± 0.25a							
			Roots	+P	1 ± 0.14c	1.51 ± 0.17b	0.89 ± 0.02c	***	***	***	***	**	***	ns
				-P	1.51 ± 0.02b	1.1 ± 0.26c	2.88 ± 0.05a							
Ribose	50	50-69-1	YS	+P	1 ± 0.03c	0.12 ± 0.03e	1.28 ± 0.09b	***	***	***	-	-	-	-
				-P	0.25 ± 0d	2.44 ± 0.05a	0.94 ± 0.09c							
			Leaves	+P	1 ± 0.01c	0.4 ± 0.02d	2.46 ± 0.53b	***	***	***	***	***	***	**
				-P	0.58 ± 0.03d	5.01 ± 0.12a	1.27 ± 0.14c							
			Roots	+P	1 ± 0.09c	0.33 ± 0.01f	1.49 ± 0.02b	***	***	***	***	***	***	ns
				-P	0.64 ± 0e	1.81 ± 0.01a	0.79 ± 0.1d							
Gulose	204	6027-89-0	YS	+P	1 ± 0.17d	1.77 ± 0.17d	4.77 ± 0.77c	***	***	***	***	***	***	ns
				-P	7.6 ± 1.59b	12.24 ± 1.41a	1.05 ± 0.04d							
			Leaves	+P	1 ± 0.04e	1.55 ± 0.28d	6.43 ± 0.6b	***	***	***	-	-	-	-
				-P	6.47 ± 0.28b	2.65 ± 0.07c	7.6 ± 0.16a							
			Roots	+P	1 ± 0.01c	9.85 ± 2.36a	1.02 ± 0.35c	***	***	***	-	-	-	-
				-P	1.01 ± 0.05c	0.91 ± 0.02c	4.27 ± 0.13b							
Galactose	204	59-23-4; 10257-28-0	YS	+P	1 ± 0.12c	11.49 ± 0.06b	2.87 ± 0.38c	***	***	***	***	***	***	ns
				-P	15.01 ± 3.2a	11.06 ± 0.1b	1.08 ± 0.05c							
			Leaves	+P	1 ± 0.22d	0.08 ± 0.04f	0.43 ± 0.07e	***	***	***	-	-	-	-
				-P	4.5 ± 0.07a	2.6 ± 0.28c	3.47 ± 0.22b							
			Roots	+P	1 ± 0.01e	2.56 ± 0.07d	1.08 ± 0.03e	***	***	***	-	-	-	-
				-P	3.79 ± 0.48c	4.28 ± 0.27b	11.75 ± 0.25a							
Galacitol	101	608-66-2	YS	+P	1 ± 0.18d	25.89 ± 1.16a	14.03 ± 0.58c	***	***	***	***	***	***	*
				-P	13.99 ± 0.08c	17.05 ± 2.52b	1.19 ± 0.1d							
			Leaves	+P	1 ± 0.01d	0.56 ± 0.23d	1.9 ± 0.05c	***	***	***	-	-	-	-
				-P	15.43 ± 0.77a	13.43 ± 0.22b	0.96 ± 0.06d							
			Roots	+P	1 ± 0.05c	1.58 ± 0.22a	1.21 ± 0.11bc	***	***	***	-	-	-	-
				-P	1.53 ± 0.11a	1.27 ± 0.01b	0.46 ± 0.15d							

Continue on the next page ...

Light intensity and P interaction effect on Primary metabolites and/or related anaplerotic pathway metabolites to carbohydrates and amino acids of Fengqing cultivar measured by GC×GC-TOF/MS analysis (Data normalise to 1 based on treatment (FL+P) (*cont.*).

Metabolites	Mass	CAS	Organ	P level	Light Intensity			Significance						
					Full Light (FL)	Medium Light (ML)	Low Light (LL)	Light (L)	P level (P)	L×P	FL	ML	LL	FL×(ML+LL)
UDP-Galactose	172	2956-16-3	YS	+P	1 ± 0.07d	2.92 ± 0.43a	2.27 ± 0.3b	***	***	***	***	***	***	***
				-P	1.54 ± 0.1c	1.67 ± 0.3c	1.47 ± 0.01c							
			Leaves	+P	1 ± 0.01d	1.33 ± 0.12b	1.17 ± 0.06c	***	***	***	-	-	-	-
				-P	0.78 ± 0.03e	2.1 ± 0.05a	0.92 ± 0d							
			Roots	+P	1 ± 0.1c	3.78 ± 0.06a	0.97 ± 0.31c	***	**	***	-	-	-	-
				-P	1.68 ± 0.11b	1.85 ± 0.04b	1.65 ± 0.32b							
Tagatose	103	87-81-0	YS	+P	1 ± 0.08c	0.6 ± 0.05d	1.6 ± 0.27b	***	***	***	-	-	-	-
				-P	2.12 ± 0.21a	2.22 ± 0.21a	2.21 ± 0.04a							
			Leaves	+P	1 ± 0.07d	1.23 ± 0.02c	2.27 ± 0.06a	***	***	***	-	-	-	-
				-P	0.95 ± 0.09d	1.43 ± 0.07b	1.21 ± 0.03c							
			Roots	+P	1 ± 0.07c	2.02 ± 0.07a	0.66 ± 0.36d	***	**	***	-	-	-	-
				-P	2.13 ± 0.16a	0.79 ± 0.07cd	1.3 ± 0.04b							
Tagatose 6P	103	67424-99-1	YS	+P	1 ± 0.28e	5.09 ± 0.04b	7.92 ± 1.09a	***	***	***	***	***	***	***
				-P	3.4 ± 0.75cd	3.85 ± 0.28c	2.47 ± 0.26d							
			Leaves	+P	1 ± 0.01e	1.43 ± 0.07c	1.36 ± 0.09cd	***	***	***	-	-	-	-
				-P	2.12 ± 0.11a	1.71 ± 0.04b	1.28 ± 0.03d							
			Roots	+P	1 ± 0.02d	3.6 ± 0.16a	1.21 ± 0.02c	***	***	***	-	-	-	-
				-P	1.36 ± 0.05b	1.42 ± 0.03b	1.07 ± 0.02d							
D-Arabitol	73	488-82-4	YS	+P	1 ± 0.5ab	0.53 ± 0.08bc	0.5 ± 0.03bc	***	**	**	-	-	-	-
				-P	1.22 ± 0.47a	1.25 ± 0.1a	0.43 ± 0.02c							
			Leaves	+P	1 ± 0ab	0.74 ± 0.01c	0.49 ± 0.18d	***	***	ns	-	-	-	-
				-P	0.91 ± 0.04b	1.08 ± 0.01a	0.2 ± 0.03e							
			Roots	+P	1 ± 0.07c	1.44 ± 0.07b	0.92 ± 0.04c	***	***	***	***	***	***	ns
				-P	1.62 ± 0.02a	0.64 ± 0.01d	1.43 ± 0.04b							
D-Xylulose	69	551-84-8	YS	+P	1 ± 0.05c	1.32 ± 0.23bc	0.97 ± 0.41c	***	***	***	***	***	**	**
				-P	2.75 ± 0.23a	0.24 ± 0d	1.54 ± 0.04b							
			Leaves	+P	1 ± 0.1e	3.64 ± 0.15c	3.58 ± 0.34c	***	***	***	***	***	***	ns
				-P	7.14 ± 0.12a	2.23 ± 0.18d	5.8 ± 0.01b							
			Roots	+P	1 ± 0.08a	0.05 ± 0.02e	0.49 ± 0.07d	***	***	ns	***	***	***	***
				-P	0.66 ± 0.15c	0.82 ± 0.02b	0.02 ± 0.01e							
D-Xylose	204	58-86-6	YS	+P	1 ± 0.09b	0.85 ± 0.04b	0.06 ± 0c	***	***	***	-	-	-	-
				-P	1.21 ± 0.18b	0.79 ± 0.02b	2.7 ± 0.64a							
			Leaves	+P	1 ± 0.1bc	1.5 ± 0.33b	0.45 ± 0.16c	***	***	***	***	-	***	ns
				-P	1.58 ± 0.04b	1.05 ± 0.02bc	4.04 ± 0.79a							
			Roots	+P	1 ± 0.02c	1.02 ± 0.02c	1.23 ± 0.05bc	***	***	***	***	***	***	ns
				-P	1.29 ± 0.02b	2.44 ± 0.34a	1.03 ± 0.05c							
Xylitol	44	87-99-0	YS	+P	1 ± 0.08b	0.62 ± 0.08c	1.98 ± 0.34a	***	***	***	-	-	-	-
				-P	0.06 ± 0.01e	0.08 ± 0.01de	0.32 ± 0.02d							
			Leaves	+P	1 ± 0.02d	2.34 ± 0.05b	3.44 ± 0.26a	***	***	*	***	***	***	***
				-P	0.11 ± 0.01e	1.67 ± 0.14c	2.23 ± 0.47b							
			Roots	+P	1 ± 0.05c	1.55 ± 0.08b	1.92 ± 0.03a	***	***	***	***	***	***	ns
				-P	0.22 ± 0.02d	0.17 ± 0.02de	0.15 ± 0.02e							
L-Xylulose	217	527-50-4	YS	+P	1 ± 0.08e	1.44 ± 0.23d	10 ± 0.21b	***	***	**	-	-	-	-
				-P	1.89 ± 0.33c	2.13 ± 0.12c	11.46 ± 0.37a							
			Leaves	+P	1 ± 0.08c	0.15 ± 0.02d	3.01 ± 0.03a	***	***	***	*	***	*	**
				-P	0.88 ± 0.07c	1.61 ± 0.19b	3.16 ± 0.12a							
			Roots	+P	1 ± 0.08a	0.26 ± 0.01bc	0.32 ± 0.01b	***	***	***	***	***	***	***
				-P	0.24 ± 0.01c	0.29 ± 0.01bc	0.29 ± 0.01bc							
L-Arabitol	217	7643-75-6	YS	+P	1 ± 0.31b	1.13 ± 0.02b	1.44 ± 0.27b	***	***	**	-	-	-	-
				-P	1.49 ± 0.5b	1.05 ± 0.01b	2.25 ± 0.31a							
			Leaves	+P	1 ± 0.02c	0.8 ± 0.02d	1.27 ± 0.02b	***	***	***	-	-	-	-
				-P	2.82 ± 0.12a	0.84 ± 0d	0.81 ± 0d							
			Roots	+P	1 ± 0.09c	2.09 ± 0.16a	0.96 ± 0.16c	***	***	**	-	-	-	-
				-P	0.71 ± 0d	1.35 ± 0.06b	0.54 ± 0.23d							
Arabinose	89	147-81-9	YS	+P	1 ± 0.51d	17.19 ± 0.15a	15.91 ± 1.25ab	***	***	***	***	ns	ns	***
				-P	11.66 ± 0.75c	17.07 ± 0.11a	15.18 ± 0.96b							
			Leaves	+P	1 ± 0.03d	0.53 ± 0.18e	1.24 ± 0.11c	***	***	***	-	-	-	-
				-P	1.84 ± 0.05a	1.54 ± 0.21b	1.13 ± 0.01cd							
			Roots	+P	1 ± 0.01d	1.87 ± 0.26a	1.24 ± 0.05c	***	**	*	-	-	-	-
				-P	1.01 ± 0.01d	1.67 ± 0.03b	1.05 ± 0.04d							
UDP-L-Arabinose	217	15839-78-8	YS	+P	1 ± 0.22a	0.16 ± 0b	0.2 ± 0.02b	***	***	***	-	-	-	-
				-P	0.11 ± 0.02b	0.12 ± 0.01b	0.08 ± 0.01b							
			Leaves	+P	1 ± 0b	0.91 ± 0.04b	1.4 ± 0.09a	***	***	***	-	-	-	-
				-P	1 ± 0b	1.37 ± 0.2a	1.32 ± 0.03a							
			Roots	+P	1 ± 0.02d	3.59 ± 0.16a	1.21 ± 0.01c	***	***	***	-	-	-	-
				-P	1.35 ± 0.05b	1.43 ± 0.05b	1.07 ± 0.02d							
UDP-L-Arabinofuranose	217	5991-05-2	YS	+P	1 ± 0.21d	13.54 ± 1.28a	7.26 ± 0.14b	***	***	***	-	-	-	-
				-P	3.14 ± 0.05c	7.02 ± 0.64b	1.47 ± 0.94d							
			Leaves	+P	1 ± 0d	1.47 ± 0.28c	0.66 ± 0.05e	***	***	***	-	-	-	-
				-P	1.58 ± 0.03bc	4.41 ± 0.2a	1.79 ± 0.09b							
			Roots	+P	1 ± 0.02cd	1.79 ± 0.18a	1.14 ± 0.04c	***	***	***	-	-	-	-
				-P	0.95 ± 0.01d	0.49 ± 0.02e	1.41 ± 0.07b							
D-Ribulose	103	488-84-6	YS	+P	1 ± 0.03c	1.91 ± 0.13b	1.25 ± 0.01c	***	***	***	-	-	-	-
				-P	3.64 ± 0.61a	1.76 ± 0.09b	1.22 ± 0.01c							
			Leaves	+P	1 ± 0.04c	0.84 ± 0.13c	1.05 ± 0.09c	***	***	***	-	-	-	-
				-P	1.38 ± 0.01b	4.37 ± 0.28a	1.32 ± 0.06b							
			Roots	+P	1 ± 0.07b	2 ± 0.35a	0.97 ± 0.05bc	***	***	***	-	-	-	-
				-P	0.7 ± 0.02d	0.74 ± 0.04cd	0.53 ± 0.03d							

Continue on the next page ...

Light intensity and P interaction effect on Primary metabolites and/or related anaplerotic pathway metabolites to carbohydrates and amino acids of Fengqing cultivar measured by GC×GC-TOF/MS analysis (Data normalise to 1 based on treatment (FL+P) (*cont.*).

Metabolites	Mass	CAS	Organ	P level	Light Intensity Full Light (FL)	Medium Light (ML)	Low Light (LL)	Significance Light (L)	P level (P)	L×P	FL	ML	LL	FL×(ML+LL)
Glycerol	117	56-81-5	YS	+P	1 ± 0.04c	5.69 ± 0.01a	4.47 ± 1.42a	***	***	***	-	-	-	-
				-P	1.3 ± 0.14bc	2.43 ± 0.94b	1.15 ± 0.16c							
			Leaves	+P	1 ± 0bc	1.65 ± 0.15a	1.15 ± 0.34b	***	***	***	-	-	-	-
				-P	0.82 ± 0.02c	0.44 ± 0.04d	1.45 ± 0.07a							
			Roots	+P	1 ± 0.11e	2.38 ± 0.17d	2.19 ± 0.02d	***	***	***	-	-	-	-
				-P	12.53 ± 0.82b	14.87 ± 0.25a	3.38 ± 0.21c							
Glycerol 3-phosphate	201	17989-41-2	YS	+P	1 ± 0.23a	0.84 ± 0.2a	0.01 ± 0b	***	***	***	-	-	-	-
				-P	0.11 ± 0.12b	0 ± 0b	0.01 ± 0b							
			Leaves	+P	1 ± 0.01a	0.06 ± 0f	0.26 ± 0e	***	***	***	-	-	-	-
				-P	0.9 ± 0.01b	0.72 ± 0.12c	0.56 ± 0.03d							
			Roots	+P	1 ± 0.05c	2.68 ± 0.03a	2.27 ± 0.49b	***	***	***	-	-	-	-
				-P	0.18 ± 0.02d	1.04 ± 0.12c	0.27 ± 0.01d							
Pectin	217	9000-69-5	YS	+P	1 ± 0.16c	1.57 ± 0.48bc	5.26 ± 0.2a	***	***	***	-	-	-	-
				-P	5.7 ± 0.9a	2.34 ± 0.7b	2.43 ± 0.87b							
			Leaves	+P	1 ± 0.01c	0.39 ± 0.31d	0.45 ± 0.01d	***	***	***	-	-	-	-
				-P	1.48 ± 0.05a	1.24 ± 0.08b	0.55 ± 0.03d							
			Roots	+P	1 ± 0.07c	12.67 ± 1.61a	1.56 ± 0.16bc	***	***	***	-	-	-	-
				-P	2.68 ± 0.43b	1.88 ± 0.12bc	2.22 ± 0.18b							
Turanose	97	547-25-1	YS	+P	1 ± 0.23c	2.77 ± 0.26a	1.61 ± 0.13b	***	***	ns	-	-	-	-
				-P	2.69 ± 0.2a	2.4 ± 0.18a	0.48 ± 0.34d							
			Leaves	+P	1 ± 0c	0.69 ± 0.02e	1.19 ± 0.02b	***	***	***	-	-	-	-
				-P	1.21 ± 0.01ab	1.29 ± 0.14a	0.81 ± 0d							
			Roots	+P	1 ± 0.08c	1.68 ± 0.01a	1.11 ± 0.01c	***	***	***	-	-	-	-
				-P	1.38 ± 0.13b	0.61 ± 0.04d	0.39 ± 0.12e							
Glucose	287	50-99-7; 2280-44-6	YS	+P	1 ± 0.23b	2.54 ± 0.07b	0.81 ± 0.3b	***	***	***	-	-	-	-
				-P	2.79 ± 0.52b	2.1 ± 0.14b	13.96 ± 2.96a							
			Leaves	+P	1 ± 0.02b	0.72 ± 0.13c	0.04 ± 0d	***	***	ns	-	-	-	-
				-P	1.69 ± 0.03a	0.09 ± 0d	0.04 ± 0d							
			Roots	+P	1 ± 0.09a	0.15 ± 0.01bc	0.17 ± 0.01b	***	***	***	-	-	-	-
				-P	0.02 ± 0d	0.08 ± 0.02cd	0.06 ± 0.01d							
L-Serine	116	56-45-1	YS	+P	1 ± 0.12c	1.54 ± 0.05b	1.64 ± 0.13b	***	*	***	***	***	***	ns
				-P	2.24 ± 0.21a	1.68 ± 0.02b	0.65 ± 0.37d							
			Leaves	+P	1 ± 0.1c	0.91 ± 0.01c	2.04 ± 0.16a	***	**	***	-	-	-	-
				-P	1.33 ± 0.08b	2 ± 0.09a	0.92 ± 0.04c							
			Roots	+P	1 ± 0.08bc	0.96 ± 0.04c	0.61 ± 0.03d	***	***	***	-	-	-	-
				-P	1.8 ± 0.14a	1.02 ± 0.11bc	1.15 ± 0.1b							
Glycine	178	56-40-6	YS	+P	1 ± 0.21c	1.77 ± 0.03ab	1.55 ± 0.64b	ns	*	***	***	***	ns	ns
				-P	2.24 ± 0.32a	1.55 ± 0.01bc	1.24 ± 0.18bc							
			Leaves	+P	1 ± 0.02d	1.32 ± 0.01cd	2.03 ± 0.46b	***	***	***	-	-	-	-
				-P	0.98 ± 0.02d	3.62 ± 0.29a	1.41 ± 0.18c							
			Roots	+P	1 ± 0.01e	1.49 ± 0.27d	2.49 ± 0.13b	***	***	***	-	-	-	-
				-P	1.93 ± 0.05c	2.9 ± 0.23a	2.11 ± 0.1c							
Threonine	102	72-19-5	YS	+P	1 ± 0.04d	7.13 ± 0.3b	0.24 ± 0.02f	***	***	***	***	***	***	ns
				-P	4.19 ± 0.12c	0.64 ± 0.01e	7.55 ± 0.15a							
			Leaves	+P	1 ± 0.03c	1.44 ± 0.01b	2.18 ± 0.16a	***	***	***	-	-	-	-
				-P	1.32 ± 0.05b	1.41 ± 0.04b	0.92 ± 0.08c							
			Roots	+P	1 ± 0.1b	1.17 ± 0.09a	1.02 ± 0.09b	**	***	**	-	-	-	-
				-P	0.92 ± 0.04bc	0.87 ± 0.06cd	0.79 ± 0.03d							
L-Homoserine	74	56-45-1	YS	+P	1 ± 0.12c	2.66 ± 0.23a	1.21 ± 0.09b	***	***	***	-	-	-	-
				-P	0.88 ± 0.03c	1.35 ± 0.02b	0.6 ± 0.02d							
			Leaves	+P	1 ± 0.41bc	0.34 ± 0d	1.51 ± 0.44b	***	***	***	-	-	-	-
				-P	4.98 ± 0.42a	0.51 ± 0.02cd	0.81 ± 0.09cd							
			Roots	+P	1 ± 0.05b	1.15 ± 0.08b	1 ± 0.09b	***	***	*	-	-	-	-
				-P	1.18 ± 0.3b	1.75 ± 0.27a	1.28 ± 0.13b							
O-Phospho-L-homoserine	68	4210-66-6	YS	+P	1 ± 0.05c	1.64 ± 0.05a	0.18 ± 0.02d	***	**	***	***	***	ns	*
				-P	1.44 ± 0.2b	0.93 ± 0.02c	0.19 ± 0d							
			Leaves	+P	1 ± 0.29d	1.43 ± 0.03c	2.68 ± 0.01a	***	***	***	-	-	-	-
				-P	1.88 ± 0.11b	0.96 ± 0.29d	0.49 ± 0.04e							
			Roots	+P	1 ± 0.03b	1.21 ± 0.08a	1 ± 0.11b	***	***	**	-	-	-	-
				-P	0.91 ± 0.06bc	0.89 ± 0.03bc	0.81 ± 0.06c							
L-Leucine	200	61-90-5	YS	+P	1 ± 0.02f	2.76 ± 0.01a	1.52 ± 0.02e	***	***	***	-	-	-	-
				-P	2.69 ± 0.02b	2.4 ± 0.01c	1.75 ± 0.02d							
			Leaves	+P	1 ± 0.01d	1.8 ± 0.01a	0.71 ± 0f	***	***	***	-	-	-	-
				-P	1.63 ± 0.02b	1.58 ± 0c	0.98 ± 0.01e							
			Roots	+P	1 ± 0.01e	1.59 ± 0.01a	1 ± 0.01e	***	***	***	-	-	-	-
				-P	1.47 ± 0.01b	1.19 ± 0.01c	1.16 ± 0d							
L-Isoleucine	158	73-32-5	YS	+P	1 ± 0.15a	0.32 ± 0.03b	0.36 ± 0.02b	***	***	***	-	-	-	-
				-P	0.21 ± 0.03c	0.08 ± 0d	0.13 ± 0.01cd							
			Leaves	+P	1 ± 0.36a	0.5 ± 0bc	1.06 ± 0.13a	***	***	ns	-	-	-	-
				-P	0.23 ± 0.03cd	0.58 ± 0.04b	0.21 ± 0.05d							
			Roots	+P	1 ± 0.01b	1.19 ± 0.07b	1.01 ± 0.09b	**	***	*	-	-	-	-
				-P	1.21 ± 0.24b	1.5 ± 0.26a	1.62 ± 0.14a							
L-Valine	142	72-18-4	YS	+P	1 ± 0.11a	0.72 ± 0.04b	0.82 ± 0.11b	***	***	ns	-	-	-	-
				-P	0.48 ± 0.11c	0.36 ± 0.11c	0.44 ± 0.06c							
			Leaves	+P	1 ± 0.05c	0.92 ± 0.01c	2.66 ± 0.12b	***	***	***	-	-	-	-
				-P	1.16 ± 0.08c	5.48 ± 0.95a	0.85 ± 0.05c							
			Roots	+P	1 ± 0.25d	1.6 ± 0.2bc	0.9 ± 0.04d	***	***	***	-	-	-	-
				-P	1.95 ± 0.12a	1.71 ± 0.2ab	1.37 ± 0.1c							

Continue on the next page ...

Light intensity and P interaction effect on Primary metabolites and/or related anaplerotic pathway metabolites to carbohydrates and amino acids of Fengqing cultivar measured by GC×GC-TOF/MS analysis (Data normalise to 1 based on treatment (FL+P) (*cont.*).

Metabolites	Mass	CAS	Organ	P level	Light Intensity Full Light (FL)	Medium Light (ML)	Low Light (LL)	Significance Light (L)	P level (P)	L×P	FL	ML	LL	FL×(ML+LL)
L-Phenylalanine	154	63-91-2	YS	+P	1 ± 0.17d	3.83 ± 0.57a	3.06 ± 0.3b	***	***	***	-	-	-	-
				-P	2.02 ± 0.13c	2.29 ± 0.3c	1.94 ± 0.02c							
			Leaves	+P	1 ± 0.2c	1.01 ± 0.01c	1.89 ± 0.14a	***	ns	ns	-	-	-	-
				-P	1.71 ± 0.2ab	1.65 ± 0.05b	0.74 ± 0.07d							
			Roots	+P	1 ± 0.12d	1.73 ± 0.04a	0.89 ± 0.04d	***	***	***	-	-	-	-
				-P	1.55 ± 0.08b	1.44 ± 0.03b	1.18 ± 0.06c							
L-Tyrosine	218	60-18-4	YS	+P	1 ± 0.01c	1.45 ± 0.05c	33.58 ± 2.6a	***	***	***	***	***	***	**
				-P	0.98 ± 0c	21.66 ± 1.22b	0.94 ± 0.35c							
			Leaves	+P	1 ± 0.3c	0.63 ± 0.01cd	1.81 ± 0.4b	***	***	ns	-	-	-	-
				-P	2.9 ± 0.18a	0.18 ± 0.02e	0.29 ± 0.03de							
			Roots	+P	1 ± 0.14c	0.9 ± 0.14c	0.69 ± 0.03d	***	***	***	-	-	-	-
				-P	1.4 ± 0.08b	2.47 ± 0.18a	1.02 ± 0.04c							
L-Tryptophan	146	73-22-3	YS	+P	1 ± 0.04de	1.43 ± 0.19b	1.17 ± 0.03cd	***	**	***	-	-	-	-
				-P	1.3 ± 0.22bc	1.93 ± 0.17a	0.85 ± 0.03e							
			Leaves	+P	1 ± 0.29d	1.43 ± 0.03c	2.68 ± 0.01a	***	***	***	-	-	-	-
				-P	1.88 ± 0.11b	0.96 ± 0.29d	0.37 ± 0.09e							
			Roots	+P	1 ± 0.12c	1.39 ± 0.22b	0.95 ± 0.11c	***	***	ns	-	-	-	-
				-P	1.68 ± 0.32ab	1.88 ± 0.06a	1.57 ± 0.05b							
L-Alanine	44	56-41-7	YS	+P	1 ± 0.03b	1.6 ± 0.04a	0.41 ± 0.02d	***	***	***	-	-	-	-
				-P	0.57 ± 0.08c	0.59 ± 0.16c	0.22 ± 0.05e							
			Leaves	+P	1 ± 0.26bc	1.08 ± 0.01b	2.22 ± 0.31a	***	***	***	-	-	-	-
				-P	0.28 ± 0d	0.76 ± 0.17c	0.17 ± 0.08d							
			Roots	+P	1 ± 0.35c	0.99 ± 0.1c	2.78 ± 0.11a	***	***	***	-	-	-	-
				-P	2.41 ± 0.03b	2.61 ± 0.01ab	2.45 ± 0.14b							
L-Asparagine	99	70-47-3	YS	+P	1 ± 0.05a	0.22 ± 0.03c	0.27 ± 0.02c	***	***	ns	*	ns	*	***
				-P	0.89 ± 0.07b	0.21 ± 0.01c	0.2 ± 0.06c							
			Leaves	+P	1 ± 0.28c	0.84 ± 0.01c	1.87 ± 0.17b	***	***	***	-	-	-	-
				-P	1.71 ± 0.06b	4.86 ± 0.44a	1.1 ± 0.11c							
			Roots	+P	1 ± 0.09bc	1.14 ± 0.07b	1 ± 0.07bc	***	***	ns	-	-	-	-
				-P	1.44 ± 0.24a	0.89 ± 0.07c	0.84 ± 0.03c							
L-Asparate	89	56-84-8	YS	+P	1 ± 0.08d	2.85 ± 0.42a	2.32 ± 0.2b	***	***	***	-	-	-	-
				-P	1.51 ± 0.1c	1.71 ± 0.22c	1.37 ± 0.06cd							
			Leaves	+P	1 ± 0.1b	2.54 ± 0.25a	2.69 ± 0.05a	***	***	***	-	-	-	-
				-P	2.49 ± 0.12a	0.63 ± 0.05c	0.99 ± 0b							
			Roots	+P	1 ± 0.06e	2.26 ± 0.09a	1 ± 0.03e	***	***	***	-	-	-	-
				-P	2.07 ± 0.14b	1.62 ± 0.08c	1.41 ± 0.12d							
Asparate 4-semialdehyde	218	15106-57-7	YS	+P	1 ± 0.03de	7.27 ± 0.09a	2.19 ± 0.29b	***	***	***	***	***	***	**
				-P	0.84 ± 0.07e	1.2 ± 0.07d	1.62 ± 0.04c							
			Leaves	+P	1 ± 0.08d	1.58 ± 0.03c	1.84 ± 0.07b	***	***	***	-	-	-	-
				-P	1.01 ± 0.06d	2.34 ± 0.17a	0.64 ± 0.03e							
			Roots	+P	1 ± 0.12c	1.18 ± 0.07b	0.98 ± 0.11c	***	***	***	-	-	-	-
				-P	1.57 ± 0.09a	1.23 ± 0.12b	1.18 ± 0.06b							
L-Glutamate	152	56-86-0	YS	+P	1 ± 0.05d	0.73 ± 0.08d	2.03 ± 0.25bc	***	***	***	***	***	***	*
				-P	1.68 ± 0.12c	2.38 ± 0.24b	5.69 ± 0.4a							
			Leaves	+P	1 ± 0.47b	0.23 ± 0.06d	0.6 ± 0.13bcd	***	***	***	**	***	***	ns
				-P	0.35 ± 0.01cd	0.7 ± 0.01bc	4.09 ± 0.31a							
			Roots	+P	1 ± 0.4bc	0.83 ± 0.03c	1.17 ± 0.07bc	***	***	**	ns	**	***	*
				-P	1.01 ± 0.1bc	1.36 ± 0.3b	1.83 ± 0.24a							
L-Glutamine	127	56-85-9	YS	+P	1 ± 0.02b	1.86 ± 0.18a	0.29 ± 0.02d	***	***	***	-	-	-	-
				-P	0.85 ± 0.06c	0.09 ± 0e	0.36 ± 0.08d							
			Leaves	+P	1 ± 0.04b	3.45 ± 0.71a	0.41 ± 0.07cd	***	***	***	***	***	***	ns
				-P	0.76 ± 0.01bc	0.2 ± 0.01d	0.65 ± 0.07bcd							
			Roots	+P	1 ± 0.01c	1.23 ± 0.18bc	1.3 ± 0.28bc	***	***	***	-	-	-	-
				-P	1.87 ± 0.17a	2.16 ± 0.1a	1.45 ± 0.17b							
Oxalic acid	57	144-62-7	YS	+P	1 ± 0.03c	1.53 ± 0.09b	0.5 ± 0.02e	***	***	***	-	-	-	-
				-P	1.09 ± 0.03c	0.85 ± 0.07d	2.34 ± 0.15a							
			Leaves	+P	1 ± 0.01d	1.82 ± 0.09a	1.25 ± 0.03b	***	***	***	-	-	-	-
				-P	1.09 ± 0.01c	0.92 ± 0e	0.65 ± 0.03f							
			Roots	+P	1 ± 0.01bc	0.82 ± 0.03e	1.04 ± 0.07b	***	**	***	-	-	-	-
				-P	0.91 ± 0.06cd	1.27 ± 0.04a	0.87 ± 0.08de							
Citrate	101	77-92-9	YS	+P	1 ± 0.05b	0.61 ± 0.07d	0.84 ± 0.01c	***	***	***	***	***	***	ns
				-P	0.63 ± 0.09d	1.06 ± 0.02ab	1.16 ± 0.07a							
			Leaves	+P	1 ± 0.05b	0.21 ± 0.03d	0.74 ± 0.06c	***	***	***	-	-	-	-
				-P	0.23 ± 0.01d	1.19 ± 0.04a	1.22 ± 0.12a							
			Roots	+P	1 ± 0.08bc	0.89 ± 0.05cd	0.81 ± 0.04d	**	***	***	-	-	-	-
				-P	0.87 ± 0.04d	1.13 ± 0.08a	1.01 ± 0.11ab							
Isocitrate	73	320-77-4; 1637-73-6	YS	+P	1 ± 0.33bc	1.85 ± 0.03a	0.87 ± 0.13c	***	***	***	ns	***	***	***
				-P	0.8 ± 0.09c	0.95 ± 0.05c	1.25 ± 0.1b							
			Leaves	+P	1 ± 0.04b	1.21 ± 0.12a	0.23 ± 0.01d	***	***	***	***	***	***	ns
				-P	0.2 ± 0.03d	0.73 ± 0.06c	1.18 ± 0.04a							
			Roots	+P	1 ± 0.06d	1.27 ± 0.08c	2.02 ± 0.23a	***	*	***	***	***	***	***
				-P	0.79 ± 0.07d	1.77 ± 0.16b	1.41 ± 0.07c							
Oxoglutarate	362	328-50-7	YS	+P	1 ± 0.11bc	0.05 ± 0.03d	1.02 ± 0.09bc	***	***	***	***	***	***	ns
				-P	1.36 ± 0.2a	1.22 ± 0.2ab	0.81 ± 0.09c							
			Leaves	+P	1 ± 0.06b	0.95 ± 0.01b	0.11 ± 0.02e	***	***	ns	***	***	***	ns
				-P	0.43 ± 0.01c	0.37 ± 0.03d	1.25 ± 0.02a							
			Roots	+P	1 ± 0.08cd	0.84 ± 0.03d	1.15 ± 0.09bc	**	***	***	***	***	*	*
				-P	1.71 ± 0.24a	1.43 ± 0.3ab	1.02 ± 0.1cd							

Continue on the next page ...

Light intensity and P interaction effect on Primary metabolites and/or related anaplerotic pathway metabolites to carbohydrates and amino acids of Fengqing cultivar measured by GC×GC-TOF/MS analysis (Data normalise to 1 based on treatment (FL+P) (*cont.*).

Metabolites	Mass	CAS	Organ	P level	Light Intensity: Full Light (FL)	Medium Light (ML)	Low Light (LL)	Significance: Light (L)	P level (P)	L×P	FL	ML	LL	FL×(ML+LL)
Succinate	147	110-15-6	YS	+P	1 ± 0.18c	1.83 ± 0.05b	2.61 ± 0.08a	***	***	***	-	-	-	-
				-P	0.56 ± 0.06d	0.11 ± 0.01e	0.26 ± 0.01e							
			Leaves	+P	1 ± 0.1c	0.43 ± 0.01e	2.1 ± 0.24a	***	***	***	***	***	***	ns
				-P	0.66 ± 0.06d	1.47 ± 0.11b	0.27 ± 0.04e							
			Roots	+P	1 ± 0.01c	1.18 ± 0.1bc	1.03 ± 0.07c	***	***	**	***	***	**	ns
				-P	1.08 ± 0.19c	1.78 ± 0.26a	1.42 ± 0.23b							
Fumarate	298	110-17-8	YS	+P	1 ± 0.05d	0.95 ± 0.02d	0.41 ± 0.02e	***	***	***	-	-	-	-
				-P	1.77 ± 0.05a	1.52 ± 0.04b	1.34 ± 0.04c							
			Leaves	+P	1 ± 0.07c	1.21 ± 0.01c	0.55 ± 0.11d	***	***	***	***	***	**	***
				-P	4.21 ± 0.25a	1.49 ± 0.08b	0.26 ± 0.11e							
			Roots	+P	1 ± 0.07c	1.19 ± 0.04bc	0.97 ± 0.07c	***	***	*	***	ns	***	ns
				-P	1.24 ± 0.1bc	1.82 ± 0.19a	1.4 ± 0.32b							
Malate	77	97-67-6	YS	+P	1 ± 0.05c	1.45 ± 0.22bc	1.71 ± 0.06b	***	***	***	-	-	-	-
				-P	12.18 ± 0.93a	0.89 ± 0.02c	1.4 ± 0.09bc							
			Leaves	+P	1 ± 0.13c	6.77 ± 0.12b	10.34 ± 3.3a	***	***	*	-	-	-	-
				-P	0.79 ± 0.06c	5.38 ± 0.22b	6.55 ± 0.57b							
			Roots	+P	1 ± 0.05b	1.12 ± 0.06a	1 ± 0.1b	***	***	ns	-	-	-	-
				-P	0.91 ± 0.06bc	0.95 ± 0.03bc	0.86 ± 0.04c							
L-Proline	116	147-85-3	YS	+P	1 ± 0.13c	9.7 ± 2.43b	0.79 ± 0.06c	***	***	***	***	***	***	ns
				-P	0.87 ± 0.02c	16.87 ± 0.04a	2.26 ± 0.32c							
			Leaves	+P	1 ± 0.02d	1.12 ± 0.01c	1.51 ± 0.01b	***	***	***	**	***	***	*
				-P	0.95 ± 0.02e	2.23 ± 0.01a	0.68 ± 0.01f							
			Roots	+P	1 ± 0.14e	13.08 ± 0.51b	1.26 ± 0.45e	***	***	***	***	***	***	***
				-P	6.92 ± 1.15d	17.73 ± 0.69a	11.44 ± 0.66c							
4-Hydroxyproline	230	51-35-4	YS	+P	1 ± 0.03a	0.35 ± 0.01d	0.59 ± 0.07c	***	***	***	***	***	***	***
				-P	0.74 ± 0.06b	0.31 ± 0.02d	0.34 ± 0.1d							
			Leaves	+P	1 ± 0.01f	1.8 ± 0.01c	4.44 ± 0.03b	***	***	***	***	***	***	ns
				-P	5.95 ± 0.05a	1.45 ± 0.02e	1.57 ± 0.02d							
			Roots	+P	1 ± 0.01c	1.31 ± 0.33bc	1.89 ± 0.08a	***	*	***	***	ns	***	ns
				-P	2.12 ± 0.11a	1.09 ± 0.29bc	1.4 ± 0.11b							
L-Arginine	179	74-79-3	YS	+P	1 ± 0.1c	1.19 ± 0.03bc	1.06 ± 0.1bc	*	***	***	-	-	-	-
				-P	1.82 ± 0.01a	1.36 ± 0.02b	1.88 ± 0.44a							
			Leaves	+P	1 ± 0.03a	0.51 ± 0.04c	0.19 ± 0.02d	***	***	***	-	-	-	-
				-P	0.91 ± 0.03b	0.89 ± 0.01b	0.91 ± 0b							
			Roots	+P	1 ± 0.91b	1.02 ± 0.07b	0.78 ± 0.07b	*	***	***	ns	**	***	ns
				-P	1.19 ± 0.22b	1.19 ± 0.06b	2.25 ± 0.12a							
Ornithine	69	70-26-8	YS	+P	1 ± 0.07b	0.92 ± 0.1b	1.84 ± 0.18a	***	***	***	-	-	-	-
				-P	0.74 ± 0.04c	0.42 ± 0.01d	0.73 ± 0.03c							
			Leaves	+P	1 ± 0.21a	0.15 ± 0.02b	0.21 ± 0.02b	***	ns	ns	-	-	-	-
				-P	0.97 ± 0.16a	0.05 ± 0b	0.13 ± 0.02b							
			Roots	+P	1 ± 0.23c	1.84 ± 0.31a	1.44 ± 0.37b	**	***	***	ns	***	**	ns
				-P	1.15 ± 0.06bc	0.94 ± 0.04c	0.94 ± 0.08c							
Histamine	154	51-45-6	YS	+P	1 ± 0.08c	1.28 ± 0.06b	0.83 ± 0.06d	***	*	***	-	-	-	-
				-P	0.05 ± 0f	2.27 ± 0.04a	0.67 ± 0.03e							
			Leaves	+P	1 ± 0.52b	0.07 ± 0c	0.15 ± 0c	***	***	***	-	-	-	-
				-P	2.5 ± 0.16a	0.75 ± 0.13b	0.04 ± 0c							
			Roots	+P	1 ± 0.09c	1.13 ± 0.11c	1.01 ± 0.1c	*	***	ns	-	-	-	-
				-P	1.25 ± 0.21bc	1.64 ± 0.34a	1.57 ± 0.27ab							
1,3,5-Trihydroxybenzene	144	87-66-1	YS	+P	1 ± 0.02c	0.56 ± 0.05d	1.52 ± 0.19b	***	***	***	-	-	-	-
				-P	1.95 ± 0.19a	2.09 ± 0.13a	2.03 ± 0.04a							
			Leaves	+P	1 ± 0.37c	1.21 ± 0c	4.06 ± 0.11a	***	***	***	-	-	-	-
				-P	1.71 ± 0.22b	1.82 ± 0.05b	0.89 ± 0.09c							
			Roots	+P	1 ± 0.11b	1.35 ± 0.19a	0.46 ± 0.02d	***	**	ns	-	-	-	-
				-P	0.88 ± 0.05bc	1.29 ± 0.21a	0.7 ± 0.02c							
L-Arogenate	218	53078-86-7	YS	+P	1 ± 0.07d	11.34 ± 0.31b	7.92 ± 0.51c	***	***	***	-	-	-	-
				-P	10.97 ± 0.14b	7.98 ± 0.22c	13.02 ± 1.44a							
			Leaves	+P	1 ± 0.19c	1.43 ± 0b	2.07 ± 0.19a	***	***	***	-	-	-	-
				-P	0.96 ± 0.13c	0.95 ± 0.17c	0.78 ± 0.05c							
			Roots	+P	1 ± 0.02b	1.17 ± 0.04b	1.03 ± 0.09b	***	***	ns	-	-	-	-
				-P	1.18 ± 0.37b	1.78 ± 0.37a	1.38 ± 0.19ab							
L-Citrulline	70	372-75-8	YS	+P	1 ± 0.05a	0.54 ± 0.05b	0.27 ± 0.02c	***	***	***	-	-	-	-
				-P	0.05 ± 0.01d	0.24 ± 0.02c	0.01 ± 0d							
			Leaves	+P	1 ± 0.53a	0.12 ± 0bc	0.46 ± 0.04b	***	***	***	-	-	-	-
				-P	0.11 ± 0.01bc	0.17 ± 0.01bc	0.06 ± 0c							
			Roots	+P	1 ± 0.17bc	1.23 ± 0.07bc	0.99 ± 0.12c	**	***	ns	-	-	-	-
				-P	1.23 ± 0.31bc	1.7 ± 0.2a	1.38 ± 0.31ab							
L-Pipecolic acid	96	3105-95-1	YS	+P	1 ± 0.04b	1.25 ± 0.17a	0.49 ± 0.15c	***	*	***	-	-	-	-
				-P	1.19 ± 0.02ab	0.58 ± 0.04c	1.23 ± 0.17a							
			Leaves	+P	1 ± 0.28b	0.55 ± 0.01c	1.26 ± 0.06b	***	***	***	-	-	-	-
				-P	2.75 ± 0.28a	0.51 ± 0.01c	0.27 ± 0.01c							
			Roots	+P	1 ± 0.14e	1.31 ± 0.27d	1.91 ± 0.08c	***	***	ns	-	-	-	-
				-P	2.99 ± 0.13a	2.73 ± 0.16a	2.32 ± 0.18b							
L-Theanine	46	3081-61-6	YS	+P	1 ± 0.07a	0.89 ± 0.52a	0.36 ± 0.03bc	***	***	ns	-	-	-	-
				-P	0.72 ± 0.07ab	0.41 ± 0.09bc	0.3 ± 0.02c							
			Leaves	+P	1 ± 0.15e	36.32 ± 0.85b	9.73 ± 2.23d	***	***	**	-	-	-	-
				-P	20.15 ± 2.64c	64.58 ± 5.72a	35.45 ± 4.29b							
			Roots	+P	1 ± 0.11cd	1.1 ± 0.04bc	0.99 ± 0.04d	***	***	***	-	-	-	-
				-P	1.33 ± 0.04a	1.1 ± 0.04bc	1.14 ± 0.03b							

Continue on the next page ...

Light intensity and P interaction effect on Primary metabolites and/or related anaplerotic pathway metabolites to carbohydrates and amino acids of Fengqing cultivar measured by GC×GC-TOF/MS analysis (Data normalise to 1 based on treatment (FL+P) (*cont.*).

Metabolites	Mass	CAS	Organ	P level	Light Intensity Full Light (FL)	Medium Light (ML)	Low Light (LL)	Significance Light (L)	P level (P)	L×P	FL	ML	LL	FL×(ML+LL)
L-Lysine	156	657-27-2	YS	+P	1 ± 0.02b	0.32 ± 0.03d	1.36 ± 0.12a	***	***	***	-	-	-	-
				-P	0.94 ± 0.01b	0.69 ± 0.13c	0.55 ± 0.2c							
			Leaves	+P	1 ± 0.04d	25.38 ± 0.52a	1.8 ± 0.11c	***	***	***	-	-	-	-
				-P	2.64 ± 0.18b	1.51 ± 0.08c	0.77 ± 0.04d							
			Roots	+P	1 ± 0.09bc	1.13 ± 0.05a	1.02 ± 0.09ab	***	***	ns	-	-	-	-
				-P	0.9 ± 0.07cd	0.95 ± 0.03bc	0.8 ± 0.02d							
Anthranilate	146	118-92-3	YS	+P	1 ± 0.07e	1.83 ± 0.01c	2.67 ± 0a	***	***	***	-	-	-	-
				-P	1.11 ± 0.02d	2.41 ± 0.04b	1.07 ± 0.03d							
			Leaves	+P	1 ± 0.01c	0.68 ± 0.01e	1.83 ± 0.01b	***	***	***	-	-	-	-
				-P	2.96 ± 0a	0.87 ± 0.01d	0.55 ± 0.01f							
			Roots	+P	1 ± 0e	1.12 ± 0.02d	0.99 ± 0.01e	***	***	***	-	-	-	-
				-P	1.31 ± 0.01c	1.84 ± 0.01a	1.56 ± 0.01b							

Means with the different letter in the row of the same metabolites content are significantly different. '***' 0.001 '**' 0.01 '*' 0.05 = significant differences, ns = non-significant differences, between light and P interaction.
'-' = no ANOVA analysis were performed. Data files from GC×GC-TOF/MS were deconvoluted using AMDIS, peaks of each chromatogram were compared to the NIST mass spectral database and published literature to identify metabolites.

Table S4. Identification of secondary metabolites measured by UPLC–Q–TOF/MS and ANOVA analysis in tea plant organs in response light effect, P effect and their interaction.

Metabolites	Mass	RT	Adduct	Mass Error (ppm)	MS Fragments	Organ	Plevel	Light Intensity			Significance						
								Full Light (FL)	Medium Light (ML)	Low Light (LL)	Light (L)	P level (P)	L×P	FL	ML	LL	FL×(ML+LL)
Quercertin	448.1006	3.32	M-H	0.06	179, 151	YS	+P	1 ± 0.22d	0.68 ± 0.1e	0.49 ± 0.09e	***	***	**	***	***	***	*
							-P	2.25 ± 0.16a	1.85 ± 0.03b	1.36 ± 0.02c							
						Leaves	+P	1 ± 0.01a	0.49 ± 0.05c	0.15 ± 0.05d	***	***	***	*	***	***	***
							-P	0.96 ± 0.04a	0.62 ± 0.02b	0.52 ± 0.02c							
						Roots	+P	1 ± 0.03a	0.4 ± 0.01e	0.3 ± 0.02f	***	***	***	***	***	***	***
							-P	0.84 ± 0.01b	0.77 ± 0.01c	0.46 ± 0d							
Isoquercetin	464.0954	4.85	M-H	8.04	301, 271, 255	YS	+P	1 ± 0.06b	0.25 ± 0.12c	0.17 ± 0.01c	***	***	***	***	***	***	***
							-P	2.34 ± 0.17a	0.14 ± 0.07c	0.21 ± 0.13c							
						Leaves	+P	1 ± 0.1e	1.55 ± 0.04b	1.25 ± 0.05d	***	***	***	***	**	***	ns
							-P	1.71 ± 0.05a	1.42 ± 0.1c	1.48 ± 0.04bc							
						Roots	+P	1 ± 0.03a	0.3 ± 0.01c	0.21 ± 0.01e	***	***	***	***	***	***	***
							-P	0.57 ± 0.02b	0.2 ± 0e	0.27 ± 0.01d							
Rutin	610.1534	4.5	M-H2O-H	5.88	593, 447, 303	YS	+P	1 ± 0.07e	2.35 ± 0.3c	1.81 ± 0.32d	***	***	***	***	***	***	ns
							-P	3.11 ± 0.48b	8.01 ± 0.19a	0.36 ± 0.01f							
						Leaves	+P	1 ± 0.04f	1.69 ± 0.06b	2.23 ± 0.04a	***	***	***	***	***	***	**
							-P	1.6 ± 0.02c	1.52 ± 0.04d	1.19 ± 0.04e							
						Roots	+P	1 ± 0.17e	4.62 ± 0.03c	3.65 ± 0.05d	***	***	***	***	***	***	ns
							-P	9.35 ± 0.45b	18.28 ± 0.27a	0.06 ± 0f							
p-Coumaryl-CoA	913.152	5.48	M+FA-H	9.99	487, 505, 565	YS	+P	1 ± 0.16b	0.73 ± 0.32b	0.82 ± 0.54b	***	*	***	-	-	-	-
							-P	0.29 ± 0.08b	3.11 ± 1.26a	0.6 ± 0.11b							
						Leaves	+P	1 ± 0.1c	1.03 ± 0.09c	0.98 ± 0.07c	***	***	ns	-	-	-	-
							-P	0.29 ± 0.13d	1.6 ± 0.09a	1.21 ± 0.06b							
						Roots	+P	1 ± 0.09b	0.17 ± 0d	1.72 ± 0.04a	***	***	***	-	-	-	-
							-P	0.1 ± 0.01de	0.05 ± 0.01e	0.28 ± 0.09c							
p-coumaryol shikimic acid	320.0896	3.73	M-H	11.70	135, 145, 164	YS	+P	1 ± 0.14a	0.02 ± 0c	0.06 ± 0.04c	***	***	***	-	-	-	-
							-P	0.24 ± 0.03b	0.26 ± 0.06b	0.13 ± 0.12bc							
						Leaves	+P	1 ± 0.1f	3.44 ± 0.19b	2.93 ± 0.12d	***	***	***	-	-	-	-
							-P	4.64 ± 0.16a	1.87 ± 0.12e	3.18 ± 0.07c							
						Roots	+P	1 ± 0.09d	2.19 ± 0.07a	0.5 ± 0.02e	***	***	***	-	-	-	-
							-P	1.61 ± 0.01c	2.03 ± 0.11b	2.04 ± 0.01b							
p-coumaroyl quinic acid	338.1002	4.48	M-H	8.91	191, 275, 293	YS	+P	1 ± 0.14b	1.2 ± 0.16b	1.93 ± 0.57a	*	***	***	-	-	-	-
							-P	2.2 ± 0.2a	2.07 ± 0.04a	1.89 ± 0.45a							
						Leaves	+P	1 ± 0.02a	0.43 ± 0.06c	0.65 ± 0.01b	***	***	***	-	-	-	-
							-P	0.1 ± 0e	1.02 ± 0.04a	0.37 ± 0.01d							
						Roots	+P	1 ± 0.02b	0.78 ± 0.06c	1.87 ± 0.04a	***	*	***	-	-	-	-
							-P	1.69 ± 0.2a	0.69 ± 0.09c	1.01 ± 0.14b							
Caffeoyl shikimic acid	336.0845	6.46	M-H2O-H	7.66	161, 179, 291	YS	+P	1 ± 0.07b	2.32 ± 1.36b	2.33 ± 0.04b	***	***	***	***	**	***	***
							-P	1.63 ± 0.07b	5.77 ± 1.45a	7.18 ± 0.85a							
						Leaves	+P	1 ± 0.03a	0.67 ± 0.03b	0.6 ± 0.03c	***	***	***	-	-	-	-
							-P	0.6 ± 0.07bc	0.51 ± 0.01d	1 ± 0.04a							
						Roots	+P	1 ± 0.03a	0.25 ± 0.01d	0.78 ± 0.01b	***	***	***	-	-	-	-
							-P	0.44 ± 0.02c	0.41 ± 0.01c	0.27 ± 0.02d							
Caffeoyl-CoA	929.1469	2.96	M-H	-11.79	109, 137, 181	YS	+P	1 ± 0.15a	0.01 ± 0b	1.23 ± 0.66a	***	***	***	-	-	-	-
							-P	0.23 ± 0.16b	0.11 ± 0.01b	0.1 ± 0.06b							
						Leaves	+P	1 ± 0.06b	0.83 ± 0.02c	0.87 ± 0.03c	***	***	***	-	-	-	-
							-P	1.09 ± 0.02a	0.67 ± 0.02e	0.74 ± 0.02d							
						Roots	+P	1 ± 0.11d	1.75 ± 0.02a	0.86 ± 0.01e	***	***	***	-	-	-	-
							-P	1.47 ± 0.01b	1.31 ± 0.01c	1.71 ± 0.02a							
Caffeoyl quinic acid	354.0951	4.91	M-H2O-H	5.92	309, 291, 179	YS	+P	1 ± 0.23d	2.64 ± 0.09b	1.66 ± 0.17c	***	***	***	-	-	-	-
							-P	2.47 ± 0.21b	1.63 ± 0.08c	5.21 ± 0.48a							
						Leaves	+P	1 ± 0.01a	0.59 ± 0.05c	0.68 ± 0.02b	***	***	***	-	-	-	-
							-P	0.25 ± 0.05d	0.99 ± 0.04a	0.64 ± 0.02bc							
						Roots	+P	1 ± 0.03a	0.36 ± 0d	0.56 ± 0.02c	*	***	***	-	-	-	-
							-P	0.05 ± 0.01e	0.77 ± 0.05b	0.5 ± 0.05c							
Naringenin	272.0685	5.05	M-H	-13.45	151, 119, 107	YS	+P	1 ± 0.08b0.05	0.53 ± 0.24b0.77	0.14 ± 0b0.5	***	***	***	-	-	-	-
							-P	0.36 ± 0.04b	9.51 ± 1.9a	0.04 ± 0.01b							
						Leaves	+P	1 ± 0.06e	2.12 ± 0.03b	1.88 ± 0.05c	***	***	***	***	***	**	**
							-P	2.08 ± 0.06b	2.65 ± 0.09a	1.71 ± 0.11d							
						Roots	+P	1 ± 0.02cd	1.21 ± 0.03b	0.93 ± 0.11d	***	***	***	***	ns	***	***
							-P	1.11 ± 0.08bc	1.89 ± 0.08a	0.9 ± 0.02d							
Eriodictyol	288.0634	4.07	M-H	5.84	151, 125, 109	YS	+P	1 ± 0.06c	1.9 ± 0.08a	0.34 ± 0.02e	**	***	***	-	-	-	-
							-P	1.66 ± 0.02b	0.6 ± 0.12d	2.04 ± 0.14a							
						Leaves	+P	1 ± 0.1cd	1.5 ± 0.1a	0.5 ± 0.06e	***	***	***	*	***	***	ns
							-P	1.12 ± 0.06bc	0.97 ± 0.07d	1.23 ± 0.05b							
						Roots	+P	1 ± 0.04a	0.22 ± 0.04c	0.02 ± 0d	***	***	***	***	ns	***	***
							-P	0.06 ± 0d	0.03 ± 0.01d	0.6 ± 0.03b							
Dihydromyricetin	320.0532	3.33	M+FA-H	-13.59	167, 195, 285	YS	+P	1 ± 0.1b	1.29 ± 0.13b	1.46 ± 0.39b	***	***	***	-	-	-	-
							-P	3.32 ± 0.52a	1.48 ± 0.45b	0.96 ± 0.27b							
						Leaves	+P	1 ± 0.12e	3.54 ± 0.14c	3.72 ± 0.15c	***	***	***	-	-	-	-
							-P	6.41 ± 0.24a	4.21 ± 0.29b	1.92 ± 0.21d							
						Roots	+P	1 ± 0.04b	0.54 ± 0.02d	0.55 ± 0.05d	***	***	***	-	-	-	-
							-P	1.62 ± 0.14a	0.83 ± 0.01c	0.23 ± 0.02e							
Myricetin	318.0376	4.4	M+FA-H	-5.22	151, 178, 271	YS	+P	1 ± 0.15b	1.83 ± 0.38a	1.01 ± 0.02b	***	***	***	-	-	-	-
							-P	0.52 ± 0.09c	0.31 ± 0.01c	1 ± 0.02b							
						Leaves	+P	1 ± 0.02a	0.82 ± 0.04b	0.54 ± 0.03c	***	***	***	***	***	ns	*
							-P	0.45 ± 0.05d	0.14 ± 0.06e	0.51 ± 0.02cd							
						Roots	+P	1 ± 0.02a	0.54 ± 0.02b	0.33 ± 0.01c	***	***	***	***	***	***	**
							-P	0.26 ± 0de	0.23 ± 0e	0.28 ± 0.02d							

Continue on the next page ...

Identification of secondary metabolites measured by UPLC–Q–TOF/MS and ANOVA analysis in tea plant organs in response light effect, P effect and their interaction (*cont.*).

Metabolites	Mass	RT	Adduct	Mass Error (ppm)	MS Fragments	Organ	Plevel	Light Intensity			Significance						
								Full Light (FL)	Medium Light (ML)	Low Light (LL)	Light (L)	P level (P)	L×P	FL	ML	LL	FL×(ML+LL)
Leucodelphinidin	322.0689	2.53	M+FA-H	-7.45	169, 197, 293	YS	+P	1 ± 0.21b	1.19 ± 0.04a	0.58 ± 0.01c	***	***	***	-	-	-	-
							-P	0.4 ± 0.02d	0.64 ± 0.02c	0.33 ± 0.01d							
						Leaves	+P	1 ± 0.04b	0.93 ± 0.03c	1 ± 0.03b	***	**	***	***	**	***	***
							-P	1.18 ± 0.05a	0.84 ± 0.04d	0.79 ± 0.02d							
						Roots	+P	1 ± 0.02a	0.86 ± 0.01b	0.77 ± 0.02c	***	***	***	***	***	ns	ns
							-P	0.51 ± 0.02d	1.01 ± 0.03a	0.77 ± 0.07c							
(+)-Gallocatechin	306.0739	2.04	M-H2O-H	2.73	137, 167, 287	YS	+P	1 ± 0.16b	3.67 ± 0.39a	0.5 ± 0.37b	**	***	ns	-	-	-	-
							-P	3.56 ± 3.13a	0.37 ± 0.32b	0.65 ± 0.19b							
						Leaves	+P	1 ± 0.04a	0.4 ± 0.01d	0.43 ± 0.02cd	***	***	***	***	***	ns	ns
							-P	0.21 ± 0e	0.54 ± 0.03b	0.48 ± 0.07bc							
						Roots	+P	1 ± 0.04d	0.51 ± 0.03e	0.99 ± 0.02d	***	***	***	***	***	***	ns
							-P	1.1 ± 0.03c	1.83 ± 0.08a	1.48 ± 0.01b							
Delphinidin	337.0149	3.9	M-H	8.55	289, 301	YS	+P	1 ± 0.32b	0.47 ± 0.01c	0.23 ± 0.01c	***	***	*	*	***	***	***
							-P	1.42 ± 0.25a	0.78 ± 0.05b	0.96 ± 0.02b							
						Leaves	+P	1 ± 0.13e	3.17 ± 0.13c	1.52 ± 0.19d	***	***	***	***	***	***	ns
							-P	5.66 ± 0.21a	4.6 ± 0.25b	4.83 ± 0.17b							
						Roots	+P	1 ± 0.02e	1.33 ± 0.05d	0.83 ± 0.01f	***	***	***	***	**	***	ns
							-P	1.82 ± 0.01a	1.4 ± 0.01c	1.58 ± 0.06b							
Delphinidin-3-glucoside	464.0955	4.86	M-H2O-H	9.83	103, 133, 179	YS	+P	1 ± 0.05d	7.52 ± 0.66a	6.36 ± 0.13bc	***	***	***	-	-	-	-
							-P	6.18 ± 0.09bc	6.9 ± 0.3ab	5.52 ± 1.18c							
						Leaves	+P	1 ± 0.01a	0.99 ± 0.02a	0.67 ± 0.03c	***	***	***	***	***	***	ns
							-P	0.68 ± 0.01c	0.76 ± 0.02b	0.44 ± 0.03d							
						Roots	+P	1 ± 0.05b	1.12 ± 0.02a	0.3 ± 0.01d	***	***	***	***	***	***	ns
							-P	0.23 ± 0.02e	0.41 ± 0.02c	0.11 ± 0.01f							
(-)-Epigallocatechin	306.0739	2.72	M+FA-H	1.67	143, 169, 289	YS	+P	1 ± 0.04bc	0.46 ± 0.26cd	0.24 ± 0.11d	***	***	***	-	-	-	-
							-P	1.45 ± 0.12b	3.87 ± 0.79a	0.41 ± 0.19cd							
						Leaves	+P	1 ± 0.2e	6.23 ± 0.44b	5.31 ± 0.2c	***	***	***	-	-	-	-
							-P	9.42 ± 0.32a	2.64 ± 0.31d	5.34 ± 0.21c							
						Roots	+P	1 ± 0.07c	0.59 ± 0e	0.58 ± 0.01e	***	***	***	-	-	-	-
							-P	0.83 ± 0.03d	1.35 ± 0.03b	1.69 ± 0.03a							
Kaempferol	286.0477	3.34	M-H	-0.30	245, 257, 269	YS	+P	1 ± 0.02a	0.31 ± 0.11c	0.23 ± 0.06c	***	***	**	***	ns	ns	***
							-P	0.87 ± 0b	0.03 ± 0.03d	0.04 ± 0.02d							
						Leaves	+P	1 ± 0.05f	1.97 ± 0.03b	1.8 ± 0.08c	*	*	***	-	-	-	-
							-P	2.2 ± 0.2a	1.22 ± 0.08e	1.58 ± 0.02d							
						Roots	+P	1 ± 0.06d	1.65 ± 0.02b	0.68 ± 0.02e	***	***	***	-	-	-	-
							-P	1.5 ± 0.03c	1.74 ± 0.01a	1.68 ± 0.01b							
Malvidin	330.047	0.81	M-H/M-H2O-H	-10.85	269, 289, 315	YS	+P	1 ± 0.55ab	0.26 ± 0.17cd	0.06 ± 0.02d	***	*	ns	ns	*	**	***
							-P	1.41 ± 0.43a	0.76 ± 0.43bc	0.02 ± 0.02d							
						Leaves	+P	1 ± 0.25e	6.36 ± 0.27c	6.18 ± 0.26c	***	***	***	-	-	-	-
							-P	11.48 ± 0.43a	7.37 ± 0.56b	2.99 ± 0.39d							
						Roots	+P	1 ± 0.03a	0.19 ± 0.01d	0.57 ± 0.03b	***	***	***	-	-	-	-
							-P	0.11 ± 0e	0.54 ± 0.05b	0.29 ± 0.01c							
Apigenin	270.0528	3.64	M-H2O-H	8.18	117, 173, 197	YS	+P	1 ± 0.06c	1.29 ± 0.08b	1.43 ± 0.26b	***	***	***	-	-	-	-
							-P	1.24 ± 0.18bc	5.18 ± 0.1a	0.7 ± 0.02d							
						Leaves	+P	1 ± 0.07f	1.52 ± 0.05c	1.65 ± 0.01b	***	**	***	***	***	***	***
							-P	1.36 ± 0.02d	1.84 ± 0.04a	1.11 ± 0.03e							
						Roots	+P	1 ± 0.05d	1.29 ± 0.08c	1.54 ± 0.02b	***	***	***	***	***	***	*
							-P	0.46 ± 0.01e	1.83 ± 0.08a	0.18 ± 0.05f							
Luteolin	286.0477	2.85	M-H	-14.48	133, 151, 175	YS	+P	1 ± 0.07bc	3 ± 1.32a	1.69 ± 0.09b	*	***	***	-	-	-	-
							-P	0.87 ± 0.07bc	0.07 ± 0.02c	1.32 ± 0.17b							
						Leaves	+P	1 ± 0.05b	1.3 ± 0.09a	0.98 ± 0.06bc	**	***	***	***	ns	***	***
							-P	0.83 ± 0.06d	0.3 ± 0.14e	0.84 ± 0.07cd							
						Roots	+P	1 ± 0.01a	0.95 ± 0.01b	0.63 ± 0.01c	***	***	***	***	***	***	ns
							-P	0.42 ± 0.01e	0.35 ± 0.02f	0.45 ± 0.01d							
Luteolin 7-*O* glucoside	462.0798	3.7	M+FA-H	-3.09	145, 287, 431	YS	+P	1 ± 0.48b	3.66 ± 1.02a	0.28 ± 0.17b	***	***	***	-	-	-	-
							-P	1.16 ± 0.79b	0.35 ± 0.27b	0.64 ± 0.89b							
						Leaves	+P	1 ± 0.01a	0.93 ± 0.02b	0.74 ± 0.01f	***	***	***	-	-	-	-
							-P	0.86 ± 0c	0.77 ± 0e	0.79 ± 0.01d							
						Roots	+P	1 ± 0.04b	0.72 ± 0.01d	0.89 ± 0.01c	***	***	***	-	-	-	-
							-P	0.75 ± 0.08d	1.61 ± 0.06a	1.06 ± 0.06b							
Leucocyanidin	306.074	2.53	M+FA-H	4.51	167, 275, 287	YS	+P	1 ± 0.11a	0.8 ± 0.05b	0.08 ± 0.01c	***	***	***	***	***	***	*
							-P	0.05 ± 0.02c	0.04 ± 0.03c	0.02 ± 0c							
						Leaves	+P	1 ± 0.22e	10.25 ± 0.39a	6.67 ± 0.46b	***	***	***	***	***	***	**
							-P	5.92 ± 0.22c	5.54 ± 0.23c	3.07 ± 0.36d							
						Roots	+P	1 ± 0.09c	0.97 ± 0.01c	2.43 ± 0.13b	***	***	***	***	***	***	ns
							-P	2.28 ± 0.17b	3.09 ± 0.08a	0.5 ± 0.11d							
Cyanidin	286.0477	3.16	2H/M+FA-H	4.87	255, 259, 283	YS	+P	1 ± 0.12bc	0.06 ± 0.02c	0.03 ± 0.04c	***	***	***	ns	***	*	ns
							-P	1.89 ± 1.35ab	2.65 ± 0.17a	0.35 ± 0.33c							
						Leaves	+P	1 ± 0.07e	2.51 ± 0.08c	1.46 ± 0.12d	***	***	***	***	ns	***	***
							-P	2.92 ± 0.16b	4.1 ± 0.12a	2.64 ± 0.11c							
						Roots	+P	1 ± 0.12b	0.01 ± 0d	0.01 ± 0d	***	***	***	ns	***	***	ns
							-P	1.01 ± 0.01b	2.93 ± 0.15a	0.59 ± 0.02c							
Cyanidin 3-glucoside	448.1006	11.66	M-H	-2.57	259, 271, 289	YS	+P	1 ± 0.09b	4.49 ± 1.39a	4.7 ± 2.42a	***	**	**	ns	***	ns	**
							-P	1.54 ± 1.23b	1.13 ± 0.01b	2.93 ± 1.6ab							
						Leaves	+P	1 ± 0.18c	1.26 ± 0.1b	1.99 ± 0.14a	***	***	***	***	ns	***	***
							-P	0.79 ± 0.14c	0.1 ± 0.01d	0.86 ± 0.19c							
						Roots	+P	1 ± 0.05e	1.74 ± 0.02b	2 ± 0.03a	***	***	***	***	***	***	**
							-P	1.34 ± 0.1d	0.97 ± 0.01e	1.57 ± 0.05c							

Continue on the next page ...

Identification of secondary metabolites measured by UPLC–Q–TOF/MS and ANOVA analysis in tea plant organs in response light effect, P effect and their interaction (*cont.*).

Metabolites	Mass	RT	Adduct	Mass Error (ppm)	MS Fragments	Organ	Plevel	Light Intensity: Full Light (FL)	Light Intensity: Medium Light (ML)	Light Intensity: Low Light (LL)	Significance: Light (L)	Significance: P level (P)	Significance: L×P	Significance: FL	Significance: ML	Significance: LL	Significance: FL×(ML+LL)
Procyanidin B1	578.1424	2.62	M-H2O-H	13.58	259, 305, 411	YS	+P	1 ± 0.27a	1.17 ± 0.21a	0.23 ± 0.07c	***	*	**	ns	***	*	**
							-P	0.95 ± 0.58ab	0.48 ± 0.12bc	0.36 ± 0.08c							
						Leaves	+P	1 ± 0.14f	7.54 ± 0.27a	2.13 ± 0.25e	***	***	***	-	-	-	-
							-P	5.17 ± 0.38b	4.72 ± 0.14c	4.28 ± 0.17d							
						Roots	+P	1 ± 0.02a	0.76 ± 0.01c	0.84 ± 0.01b	***	***	*	-	-	-	-
							-P	0.74 ± 0.03c	0.5 ± 0.02e	0.62 ± 0.01d							
Chalcone	208.0888	4.13	M+Na-2H	-5.61	103, 105, 131	YS	+P	1 ± 0.13b	0.42 ± 0.23cd	0.23 ± 0.01d	***	***	ns	-	-	-	-
							-P	1.29 ± 0.07a	0.56 ± 0.04c	0.44 ± 0.12c							
						Leaves	+P	1 ± 0.02a	0.45 ± 0.05d	0.54 ± 0.03c	***	***	***	-	-	-	-
							-P	0.15 ± 0.06e	0.82 ± 0.04b	0.52 ± 0.02cd							
						Roots	+P	1 ± 0.03a	0.3 ± 0d	0.69 ± 0.02b	***	***	***	-	-	-	-
							-P	0.51 ± 0.01c	0.33 ± 0.03d	0.24 ± 0.02e							

Means with the different letter in the row of the same metabolites content are significantly different. '***' 0.001 '**' 0.01 '*' 0.05 = significant differences, ns = non-significant differences, between light and P interaction.'-' = no ANOVA analysis were performed. The metabolites were identified based on actual mass, retention time and isotopic distribution and accurate mass measurements were confirmed from Metlin online web based database and published literature.

Table S5. Targeted metabolites amino acid and catechins (mg g^{-1}) in young shoots and leaves of tea plants in response light effect, P effect and their interaction effect.

	Organ	P level	Light Intensity			Significance		
Metabolites			FL	ML	LL	light	Plevel	L×P
Amino acids								
Ser	Young Shoots	+P	0.19 ± 0.01a	0.12 ± 0.01c	0.12 ± 0.02c	$p<0.001$	$p<0.001$	$p<0.001$
		-P	0.15 ± 0.01b	0.14 ± 0bc	0.09 ± 0d			
	Leaves	+P	0.36 ± 0.06a	0.15 ± 0.03c	0.12 ± 0.03c	$p<0.001$	$p<0.01$	$p<0.01$
		-P	0.24 ± 0.08b	0.08 ± 0.02c	0.16 ± 0.03bc			
Gly	Young Shoots	+P	2.14 ± 0.4a	0.72 ± 0.11b	0.54 ± 0.08b	$p<0.001$	$p<0.001$	$p<0.001$
		-P	0.74 ± 0.12b	0.62 ± 0.12b	0.49 ± 0.05b			
	Leaves	+P	2.17 ± 0.86a	0.2 ± 0.06c	0.47 ± 0.3bc	$p<0.001$	$p<0.001$	$p<0.001$
		-P	0.91 ± 0.27b	0.11 ± 0.06c	0.35 ± 0.11bc			
Thr	Young Shoots	+P	0.38 ± 0.01c	0.43 ± 0b	0.28 ± 0.01f	$p<0.001$	$p<0.001$	$p<0.001$
		-P	0.35 ± 0d	0.33 ± 0.01e	0.45 ± 0a			
	Leaves	+P	0.49 ± 0.01c	0.22 ± 0.01f	0.69 ± 0.01b	$p<0.001$	$p<0.001$	$p<0.001$
		-P	0.3 ± 0.01d	0.79 ± 0.01a	0.26 ± 0.01e			
Ile	Young Shoots	+P	0.53 ± 0.01c	0.64 ± 0.01a	0.66 ± 0.02a	$p<0.001$	$p<0.001$	$p<0.001$
		-P	0.58 ± 0.01b	0.36 ± 0.01e	0.49 ± 0.01d			
	Leaves	+P	0.18 ± 0.05c	0.6 ± 0.2a	0.39 ± 0.09b	$p<0.001$	$p<0.001$	$p<0.001$
		-P	0.19 ± 0.05c	0.1 ± 0.03c	0.09 ± 0.03c			
Phe	Young Shoots	+P	1.17 ± 0.02b	1.22 ± 0.01a	1.14 ± 0.01c	$p<0.001$	$p<0.001$	$p<0.001$
		-P	1.06 ± 0.02d	1.08 ± 0.01d	1.04 ± 0.01e			
	Leaves	+P	0.45 ± 0.16bc	0.79 ± 0.2b	1.32 ± 0.37a	$p<0.001$	$p<0.001$	$p<0.01$
		-P	0.51 ± 0.16bc	0.27 ± 0.07c	0.77 ± 0.14b			
Val	Young Shoots	+P	0.61 ± 0.01c	0.76 ± 0.03a	0.78 ± 0.02a	$p<0.01$	$p<0.001$	$p<0.001$
		-P	0.68 ± 0.01b	0.5 ± 0.01e	0.54 ± 0.01d			
	Leaves	+P	0.09 ± 0.02b	0.17 ± 0.01a	0.15 ± 0.02a	$p<0.001$	$p<0.001$	$p<0.001$
		-P	0.09 ± 0.01b	0.08 ± 0b	0.08 ± 0.01b			
Ala	Young Shoots	+P	0.12 ± 0.01c	0.21 ± 0.01a	0.11 ± 0.02c	$p<0.001$	$p<0.01$	$p<0.01$
		-P	0.12 ± 0.01c	0.18 ± 0.01b	0.1 ± 0.01c			
	Leaves	+P	0.07 ± 0.01b	0.02 ± 0.01c	0.09 ± 0.02a	$p<0.001$	$p<0.001$	$p<0.001$
		-P	0.02 ± 0.01c	0.01 ± 0.01c	0.08 ± 0.02ab			
Asp	Young Shoots	+P	3.69 ± 0.21a	3.15 ± 0.02b	3.21 ± 0.02b	$p<0.001$	$p<0.001$	$p<0.001$
		-P	3.34 ± 0.18b	2.83 ± 0.03c	1.67 ± 0.14d			
	Leaves	+P	1.99 ± 0.56	1.53 ± 0.24ab	1.02 ± 0.29bc	$p<0.001$	$p<0.001$	$p<0.01$
		-P	1.8 ± 0.49	0.33 ± 0.06d	0.83 ± 0.2cd			
Glu	Young Shoots	+P	4.41 ± 0.01e	4.26 ± 0.01f	4.64 ± 0.01c	$p<0.001$	$p<0.001$	$p<0.001$
		-P	4.5 ± 0.01d	4.86 ± 0.03b	5.51 ± 0.01a			
	Leaves	+P	2.68 ± 0.51bcd	2.91 ± 0.86bc	1.72 ± 0.46d	$p<0.001$	$p<0.001$	$p<0.001$
		-P	2.34 ± 0.31cd	4.87 ± 1.01a	3.62 ± 0.49b			
Pro	Young Shoots	+P	3.75 ± 0.04e	5.33 ± 0.05b	3.44 ± 0.28e	$p<0.001$	$p<0.001$	$p<0.001$
		-P	4.17 ± 0.03d	5.96 ± 0.18a	4.75 ± 0.32c			
	Leaves	+P	0.94 ± 0.32d	0.84 ± 0.38d	5.11 ± 1.22b	$p<0.001$	$p<0.001$	$p<0.001$
		-P	1.17 ± 0.75cd	2.86 ± 0.59c	10.19 ± 1.73a			
Arg	Young Shoots	+P	1.03 ± 0.04b	0.8 ± 0c	0.31 ± 0.01d	$p<0.001$	$p<0.001$	$p<0.001$
		-P	1.06 ± 0.05b	1 ± 0.08b	1.56 ± 0.03a			
	Leaves	+P	1.35 ± 0.39b	0.46 ± 0.29b	0.58 ± 0.17b	$p<0.001$	$p<0.001$	$p<0.001$
		-P	3.48 ± 0.66a	4.78 ± 1.99a	0.67 ± 0.16b			
Cys	Young Shoots	+P	0.42 ± 0.01d	1.1 ± 0.04a	0.62 ± 0.01b	$p<0.001$	$p<0.001$	$p<0.001$
		-P	0.64 ± 0.01b	0.64 ± 0.01b	0.53 ± 0.03c			
	Leaves	+P	1.55 ± 0.28a	1.01 ± 0.25b	0.5 ± 0.2c	$p<0.001$	$p<0.001$	$p<0.001$
		-P	0.35 ± 0.08cd	0.19 ± 0.08cd	0.09 ± 0.03d			
His	Young Shoots	+P	0.37 ± 0.09b	0.81 ± 0.2a	0.93 ± 0.29a	$p<0.05$	$p<0.001$	$p<0.001$
		-P	0.98 ± 0.29a	0.15 ± 0.03b	0.15 ± 0.02b			
	Leaves	+P	0.15 ± 0.03bc	0.25 ± 0.05b	0.13 ± 0.03bc	$p<0.001$	$p<0.001$	$p<0.001$
		-P	0.09 ± 0.03c	0.88 ± 0.19a	0.09 ± 0.02c			
Leu	Young Shoots	+P	0.75 ± 0.01b	0.86 ± 0.01a	0.49 ± 0.01e	$p<0.001$	$p<0.001$	$p<0.001$
		-P	0.65 ± 0c	0.53 ± 0.01d	0.52 ± 0.01d			
	Leaves	+P	0.24 ± 0.01c	0.05 ± 0f	0.51 ± 0.01a	$p<0.001$	$p<0.001$	$p<0.001$
		-P	0.42 ± 0.01b	0.1 ± 0e	0.13 ± 0.01d			
Lys	Young Shoots	+P	1.03 ± 0.02b	1.03 ± 0.01b	1.04 ± 0.03b	$p<0.001$	$p<0.001$	$p<0.001$
		-P	1.18 ± 0.01a	1.21 ± 0.01a	1 ± 0.06b			
	Leaves	+P	0.52 ± 0.1bc	1.26 ± 0.11a	0.7 ± 0.19b	$p<0.001$	$p<0.001$	$p<0.001$
		-P	0.47 ± 0.1cd	0.29 ± 0.09d	0.44 ± 0.11cd			
Met	Young Shoots	+P	4.39 ± 1.36b	4.73 ± 0.97b	5.38 ± 1.01b	$p<0.001$	$p<0.001$	$p<0.001$
		-P	11.62 ± 2.54a	4.15 ± 0.81b	3.92 ± 0.63b			
	Leaves	+P	2.22 ± 0.52a	1.88 ± 0.44a	1.05 ± 0.16b	$p<0.001$	$p<0.001$	$p<0.001$
		-P	0.41 ± 0.12c	1.12 ± 0.25b	0.1 ± 0.03c			
Thea	Young Shoots	+P	59.81 ± 3.28a	35.51 ± 3.12c	33.98 ± 2.25c	$p<0.001$	$p<0.001$	$p<0.001$
		-P	49.12 ± 3.25b	61.76 ± 1.6a	31.65 ± 1.34c			
	Leaves	+P	20.99 ± 0.47b	15.49 ± 0.26d	28.5 ± 0.33a	$p<0.001$	$p<0.001$	$p<0.001$
		-P	17.84 ± 0.3c	10.66 ± 0.47e	17.16 ± 0.56c			
Tyr	Young Shoots	+P	0.27 ± 0.01d	0.57 ± 0.01c	1.76 ± 0.02a	$p<0.001$	$p<0.001$	$p<0.001$
		-P	0.21 ± 0.01e	0.99 ± 0.05b	0.06 ± 0.02f			
	Leaves	+P	0.28 ± 0.07cd	1.65 ± 0.44a	0.58 ± 0.18c	$p<0.001$	$p<0.001$	$p<0.001$
		-P	0.21 ± 0.06cd	0.06 ± 0.05d	0.98 ± 0.23b			

Continue on the next page...

Targeted metabolites amino acid and catechins (mg g^{-1}) in young shoots and leaves of tea plants in response light effect, P effect and their interaction effect. (*cont.*).

Metabolites	Organ	P level	Light Intensity FL	ML	LL	Significance light	Plevel	L×P
Catechins								
EGCG	Young Shoots	+P	45.69 ± 1.58b	38.5 ± 2.67c	37.23 ± 1.42c	$p<0.001$	$p<0.001$	$p<0.01$
		-P	50.75 ± 1.02a	38.49 ± 0.9c	38.25 ± 1.26c			
	Leaves	+P	31.44 ± 6.04ab	23.06 ± 4.85bc	32.7 ± 8.96ab	$p<0.01$	$p<0.01$	ns
		-P	37.73 ± 9.83a	29.77 ± 3.86abc	19.21 ± 5.52c			
C	Young Shoots	+P	1.36 ± 0.02e	1.38 ± 0.01e	2.04 ± 0.01b	$p<0.001$	$p<0.001$	$p<0.05$
		-P	1.48 ± 0.01d	1.54 ± 0.02c	2.16 ± 0.03a			
	Leaves	+P	0.2 ± 0.02a	0.16 ± 0.03abc	0.11 ± 0.02c	$p<0.001$	$p<0.05$	ns
		-P	0.16 ± 0.03ab	0.12 ± 0.03bc	0.14 ± 0.03bc			
CG	Young Shoots	+P	0.11 ± 0a	0.06 ± 0.01c	0.03 ± 0d	$p<0.001$	$p<0.001$	$p<0.001$
		-P	0.08 ± 0.01b	0.05 ± 0c	0.05 ± 0c			
	Leaves	+P	0.09 ± 0a	0.04 ± 0e	0.05 ± 0d	$p<0.001$	$p<0.001$	$p<0.001$
		-P	0.07 ± 0b	0.03 ± 0f	0.06 ± 0c			
EC	Young Shoots	+P	1.14 ± 0.01a	0.96 ± 0.01c	0.9 ± 0d	$p<0.001$	$p<0.001$	$p<0.001$
		-P	1.02 ± 0.01b	0.96 ± 0.01c	0.87 ± 0.02e			
	Leaves	+P	0.26 ± 0.02a	0.23 ± 0.01b	0.21 ± 0.01c	$p<0.001$	$p<0.001$	ns
		-P	0.2 ± 0c	0.21 ± 0.01c	0.23 ± 0.01b			
ECG	Young Shoots	+P	3.62 ± 0.02b	3.61 ± 0.01b	2.58 ± 0.05c	$p<0.001$	$p<0.001$	$p<0.001$
		-P	3.77 ± 0.03a	2.3 ± 0e	2.49 ± 0.04d			
	Leaves	+P	3.61 ± 1ab	0.69 ± 0.09c	2.07 ± 0.86bc	$p<0.001$	$p<0.05$	ns
		-P	4.86 ± 1.86a	1.85 ± 0.4c	2.18 ± 0.51bc			
GA	Young Shoots	+P	0.93 ± 0.02b	0.9 ± 0c	0.84 ± 0.01e	$p<0.001$	$p<0.001$	$p<0.001$
		-P	0.97 ± 0.02a	0.88 ± 0.01d	0.76 ± 0.01f			
	Leaves	+P	0.46 ± 0.01a	0.44 ± 0.01ab	0.28 ± 0.03d	$p<0.001$	$p<0.001$	$p<0.001$
		-P	0.45 ± 0.02a	0.4 ± 0c	0.41 ± 0.01bc			
GCG	Young Shoots	+P	0.45 ± 0b	0.54 ± 0.02a	0.53 ± 0.02a	$p<0.001$	$p<0.001$	$p<0.001$
		-P	0.25 ± 0.01d	0.27 ± 0.02d	0.33 ± 0.01c			
	Leaves	+P	0.05 ± 0c	0.04 ± 0d	0.02 ± 0f	$p<0.001$	$p<0.001$	$p<0.001$
		-P	0.09 ± 0a	0.03 ± 0e	0.06 ± 0b			

Means with different letters in the same row of the same metabolite are significantly different. ns = non-significant differences, between light and P interaction.
